# Efficient genome editing in filamentous fungi via an improved CRISPR‐Cas9 ribonucleoprotein method facilitated by chemical reagents

**DOI:** 10.1111/1751-7915.13652

**Published:** 2020-08-25

**Authors:** Gen Zou, Meili Xiao, Shunxing Chai, Zhihua Zhu, Ying Wang, Zhihua Zhou

**Affiliations:** ^1^ CAS‐Key Laboratory of Synthetic Biology CAS Center for Excellence in Molecular Plant Sciences Institute of Plant Physiology and Ecology Chinese Academy of Science Fenglin Rd 300 Shanghai 200032 China; ^2^ Shanghai Key Laboratory of Agricultural Genetics and Breeding Institute of Edible Fungi Shanghai Academy of Agriculture Science 1000 Jinqi Rd, Fengxian Shanghai 201403 China; ^3^ University of Chinese Academy of Sciences Beijing 100049 China

## Abstract

DNA double‐strand break (DSB) repair induced by the RNA‐programmed nuclease Cas9 has become a popular method for genome editing. Direct genome editing via Cas9‐CRISPR gRNA (guide RNA) ribonucleoprotein (RNP) complexes assembled *in vitro* has also been successful in some fungi. However, the efficiency of direct RNP transformation into fungal protoplasts is currently too low. Here, we report an optimized genome editing approach for filamentous fungi based on RNPs facilitated by adding chemical reagents. We increased the transformation efficiency of RNPs significantly by adding Triton X‐100 and prolonging the incubation time, and the editing efficiency reached 100% in *Trichoderma reesei* and *Cordyceps militaris*. The optimized RNP‐based method also achieved efficient (56.52%) homologous recombination integration with short homology arms (20 bp) and gene disruption (7.37%) that excludes any foreign DNA (selection marker) in *T. reesei*. In particular, after adding reagents related to mitosis and cell division, the further optimized protocol showed an increased ratio of edited homokaryotic transformants (from 0% to 40.0% for inositol and 71.43% for benomyl) from *Aspergillus oryzae,* which contains multinucleate spores and protoplasts. Furthermore, the multi‐target engineering efficiency of the optimized RNP transformation method was similar to those of methods based on *in vivo* expression of Cas9. This newly established genome editing system based on RNPs may be widely applicable to construction of genome‐edited fungi for the food and medical industries, and has good prospects for commercialization.

## Graphical abstract

We report an optimized genome editing approach for filamentous fungi based on RNPs facilitated by adding chemical reagents. We increased the gene editing efficiency of RNPs significantly by adding Triton X‐100. Reagents related to mitosis and cell division furtherly increased ratio of edited homokaryotic transformants from filamentous fungus containing multinucleate spores and protoplasts.

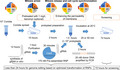

## Introduction

The CRISPR‐Cas9 system has proven to be compatible with nearly all species from prokaryotes to eukaryotes with various modifications (DiCarlo *et al*., 2013; Auer *et al*., 2014; Copeland *et al*., 2014; Yang *et al*., 2014; Liu *et al*., 2015). Usually, researchers introduce the Cas9 and sgRNA cassette with appropriate promoters into cells using plasmids to produce a functional Cas9/sgRNA ribonucleoprotein (Ran *et al*., 2013). Due to use of the prokaryote‐derived Cas9 protein, the coding gene requires codon optimization for eukaryotic cells in most cases (Hruscha *et al*., 2013; Liu *et al*., 2015; Nødvig *et al*., 2015). This requirement has prevented implementation of the CRISPR‐Cas9 system in organisms that lack genomic sequences and codon usage databases (http://www.kazusa.or.jp/codon/). Moreover, the introduction of the Cas9 coding gene using classic transgenic technology might affect genome structure. In addition, it has been frequently reported that retaining Cas9 expression *in vivo* may cause unexpected phenotypes, such as delayed growth (Enkler *et al*., 2016), further rearrangements or off‐target mutations (Jacobs *et al*., 2014) and even toxic effects in host cells (Cho *et al*., 2017; Jiang *et al*., 2017). Cas9 could be transiently expressed from a non‐replicating plasmid introduced to the protoplasts and presumably not integrated into the genome (Arazoe *et al*., 2015; Matsu‐ura *et al*., 2015). However, transfected plasmids degrade within cells due to endogenous nucleases, and the resulting small DNA fragments may be inserted at both on‐target and off‐target sites in host cells (Kim *et al*., 2014).

Recently, CRISPR/Cas9 RNP‐mediated genome editing systems have been widely applied in animals and other organisms. This strategy has a distinct advantage over *in vivo* expression of Cas9 and gRNA, as Cas9‐gRNA RNP assembly is not limited by the amount or rate of Cas9 translation or gRNA transcription and the gRNA may be protected from degradation. In addition, genome editing through the delivery of RNPs reduces unwanted gene targeting and avoids integrational mutagenesis, which may occur due to gene delivery strategies (Lin *et al*., 2014; Ramakrishna *et al*., 2014; Zuris *et al*., 2015). In animal embryos, microinjection technology can easily introduce pre‐assembled RNPs into cells. By injecting highly pure, pre‐assembled and optimally solubilized Cas9‐sgRNA RNPs into embryos, the mutagenesis efficiency became exceptionally high and mutagenesis rates reached 100% for individual target loci, generating complete somatic mutants (Burger *et al*., 2016).

Not all cells and organisms can be microinjected in the same manner as embryonic cells. Direct and efficient delivery of Cas9‐RNP into the cytosol followed by translocation to the nucleus remains a challenge. A few strategies for Cas9 protein delivery, such as electroporation (Kim *et al*., 2014), membrane deformation (Lau and Hamer, 1998) and even nanoparticle (Kim *et al*., 2014; Mout *et al*., 2017), have been reported in animals. However, these strategies require specialized processing techniques and are generally impractical for *in vivo* therapeutic application. In plants, PEG‐mediated protoplast transfection successfully delivered RNPs into the protoplasts of various plant species (Woo *et al*., 2015; Kanchiswamy, 2016). PEG‐mediated fungal protoplast transformation is also a routine strategy used to introduce DNA and RNA into fungi (Liu *et al*., 2015). As in plants, the main steps of the CRISPR/Cas9 RNP‐mediated genome editing system based on PEG‐mediated protoplast transformation in fungi include RNP preparation, RNP delivery, protoplast regeneration and mutant identification. However, fungal protoplasts (2–5 μm) are much smaller than those of plants (10–100 μm). Therefore, much less RNPs may be adsorbed on the surface of fungal protoplasts compare to those of plants. Due to the low ratio of RNPs delivered into *Penicillium chrysogenum* protoplasts, the genome editing efficiency of RNPs was reported to be extremely low (1% in wild strain; Pohl *et al*., 2016). A recent report also showed low genome editing efficiency through classic protoplast transformation with RNP in *T*. *reesei* (Hao and Su, 2019). For *cel3c* deletion, only five of 143 transformants were repaired through homology‐directed repair (HDR), which was quite similar to the deletion efficiency of traditional genetic manipulation without CRISPR/ Cas9 (Arentshorst *et al*., 2012; Hao and Su, 2019).

To reduce ectopic integration events, NHEJ‐deficient background (such as ∆*ku80*, *∆kusA* and *∆ligD*) strains are generally used to improve the editing efficiency of RNP‐based methods (Pohl *et al*., 2016; Kwon *et al*., 2019; van Leeuwe *et al*., 2019). With a ‘pre‐edited’ chassis, single and multiple gene deletion using RNPs increased to 10%˜100% in *Thermothelomyces thermophilus* (Kwon *et al*., 2019), while single gene deletion using RNP increased to 66.7˜100% in *Aspergillus niger* (van Leeuwe *et al*., 2019), and to 33.3˜100% in *P. chrysogenum* (Pohl *et al*., 2016). After genome editing, recycling of the selectable marker is a viable method for attaining marker‐free transformation. However, Δ*ku* strains were still screened using transgenes as selectable markers. In addition, Δ*ku* strains of some fungal species are more sensitive to specific chemicals in the growth environment, such as phleomycin, bleomycin and methyl/ethyl methanesulfonate (van Attikum *et al*., 2001; Liu *et al*., 2015). In summary, there is no report on enhancing the editing efficiency via increasing the RNPs penetration or via controlling the mitotic cycle in fungi.

In this study, we used the surfactant Triton X‐100, which improves cell membrane permeability, to increase RNP entry into protoplasts. In addition, inositol and benomyl were employed to control cell division and the mitotic cycle. An efficient CRISPR/Cas9 system based on RNPs was established benefitting from these chemical reagents in filamentous fungi *Trichoderma reesei*, *Cordyceps militaris* and *Aspergillus oryzae,* which are widely applied in the food and feed industries. This system allowed us to introduce mutagenesis at a target site in the fungal genome without leaving any introduced DNA. It can also disrupt genes with reduced side‐effects on the genome compared with those caused by introduction of the Cas9 expression cassette.

## Results

### Optimization of polyethylene glycol (PEG)‐mediated protoplast transformation of RNPs in *T. reesei*

With appropriately designed gRNAs, the genome can be cut at any target location by Cas9 protein. As *ura5* has a negative selection effect, growth inhibition can be observed in minimal medium (MM) plates containing 5‐fluoro‐orotic acid (5‐FOA; cells with wild‐type URA5 convert 5‐FOA into the toxic substance 5ʹ fluorouridine monophosphate). In *T*. *reesei* Rut‐C30, 1.5 mg ml^−1^ 5‐FOA completely inhibits protoplast regeneration and mycelial growth (Liu *et al*., 2015). Therefore, to test the efficiency of protoplast transformation of RNPs in this study, gRNA (gRNA_*Trura5*, template: gRNA_M_*Trura5*; Table S1) was designed to target *Trura5* (Trire2|21435) and then assembled with the recombinant Cas9 nuclease using a commercial Cas9 kit to form RNPs. To determine the effect of the RNP concentration on the targeting efficiency, RNPs at a range of concentrations (0, 20, 60, 100 and 170 nM) were transformed into *T. reesei* protoplasts (˜2 × 10^5^ protoplasts) via PEG‐mediated transformation. After 3 days incubation on MM plates containing 5‐FOA, the resulting number of colony‐forming units (CFUs) was determined and the corresponding efficiency was analysed by PCR and sequencing using the Trura5F and Trura5R primers (Table S1; Fig. S1). No *T. reesei* transformants were obtained from transformations without RNPs or with RNPs at concentrations ≤ 20 nM. As the RNP concentration increased, the resulting number of CFUs also increased (Table 1). In total, only nine *T. reesei* transformants (1 CFU at 60 nM, 2 CFUs at 100 nM and 6 CFUs at 170 nM) were obtained through the standard protoplast transformation procedure of RNP; their *ura5* genes were sequenced. Eight of the transformants (2 CFUs at 100 nM and 6 CFUs at 170 nM) exhibited the classic mutations in the vicinity of the cleavage site. Even with a high RNP concentration of 170 nM, the observed number of CFUs (6 CFUs per 2 × 10^5^ protoplasts) obtained through standard PEG‐mediated transformation was quite low in *T. reesei*, most likely due to the difficulty of RNP entry into cells.

**Table 1:** CRISPR/Cas9‐based mutagenesis on *Trura5* using different procedures.

RNP (nM)	Standard procedure		Optimized procedure	
	CFUs	Positive rate	CFUs	Positive rate
0	0	0	0	0
20	0	0	0	0
60	1(0/1)^a^	0	2 (1/2)	50.00%
100	2(2/2)	100.00%	8(8/8)	100.00%
170	6(6/6)	100.00%	26 (12/12)	100.00%

^a^ Positive CFU(s)/sequenced CFU(s).

To ensure that RNPs cross the fungal cell membrane and nuclear membrane successfully during PEG‐mediated protoplast transformation, the surfactant Triton X‐100 was used to improve cell membrane permeability (Ahyayauch *et al*., 2012; Mattei *et al*., 2017). Protoplast solutions with different amounts of RNPs were mixed with Triton X‐100 [0.006% (w/v) final concentration in transformation reaction], and the incubation time (at 20°C) was prolonged to 25 min before the mixture was transferred to the selective medium. The optimized transformation procedure resulted in a significant increase (3.33‐fold) in CFUs (Table 1). The efficiency of RNP transformation into *T. reesei* reached 26 CFUs per 2 × 10^5^ protoplasts with 170 nM RNPs, which was similar to that of the standard PEG‐mediated DNA transformation and provided sufficient CFUs for further genome editing and analyses. With the increased transformation efficiency, 100% of randomly selected *T. reesei* transformants showed a mutation at the target site located proximal to the upstream of the protospacer adjacent motif (PAM) sequence (Fig. S1) using high RNP concentrations (100 and 170 nM). This finding is consistent with CRISPR/Cas9‐guided DSBs generally being repaired through the process of NHEJ, which generates mutations in the vicinity of the cleavage site.

The *Trura5* genes of 31 selected *T. reesei* transformants (all nine obtained through the standard RNP transformation procedure and 22 selected from 36 CFUs generated by the optimized procedure) were sequenced, most of which exhibited the classic mutations in the vicinity of the cleavage site (Table 1). Only two of the sequenced *T. reesei* transformants lacked mutations at the *Trura5* gene. These two transformants were obtained through both the standard and optimized RNP transformation procedures with low RNP concentration (60 nM).

### Comparative analysis of RNP‐directed and other URA5 mutageneses in *T. reesei*

**Fig. 1:**
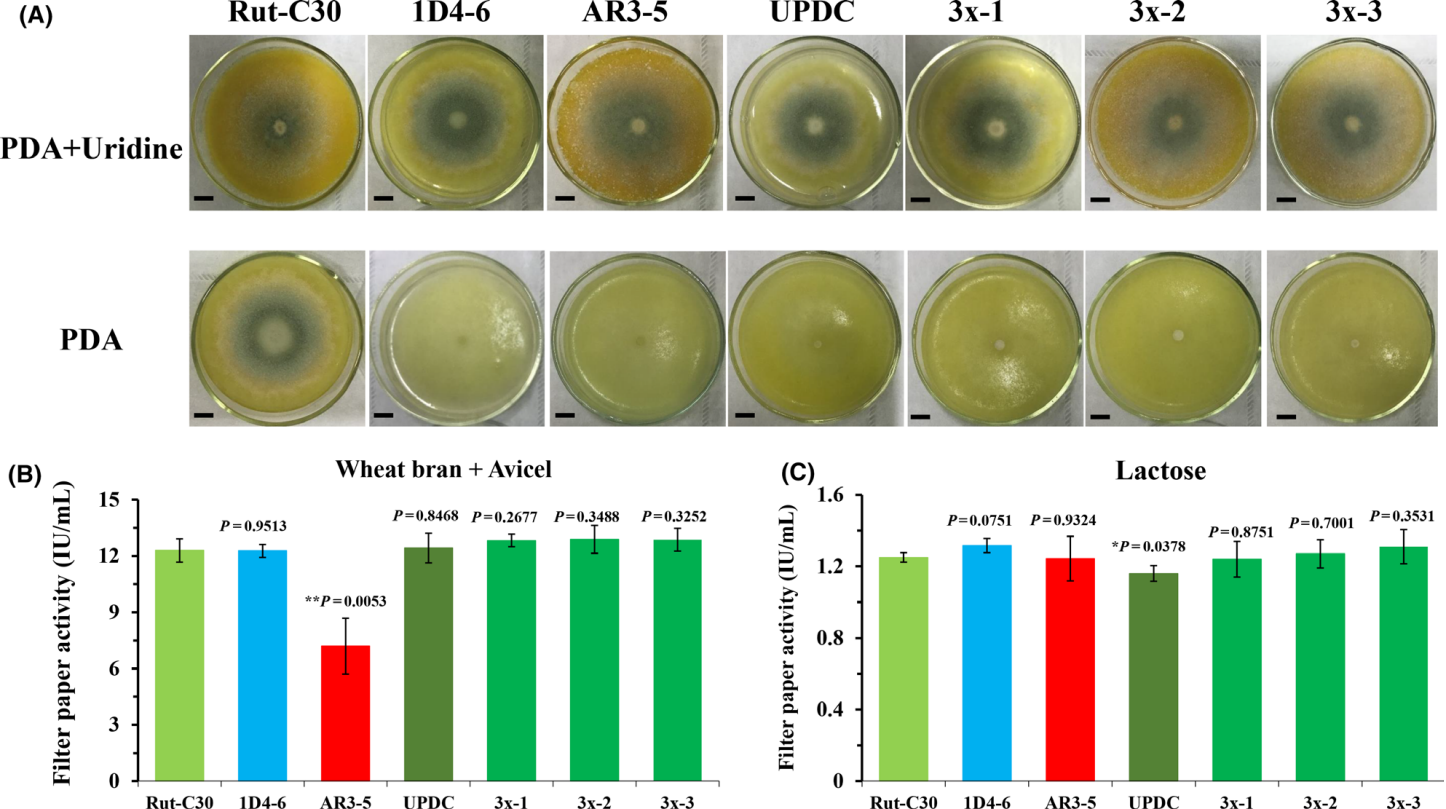
Comparative analysis of *ura5*‐deficient mutants obtained through different methods. A. Phenotype of Rut‐C30 and its *Trura5‐*deficient mutants on PDA plates (with/without uridine). Rut‐C30: wild type. 1D4–6: *Trura5‐*deficient mutant (UV). AR3–5: *Trura5‐*deficient mutant (edited with pcbh1 promoter‐controlled Cas9). UPDC: *Trura5‐*deficient mutant (edited with ppdc promoter‐controlled Cas9). 3x–1 ˜ 3x–3: *Trura5‐*deficient mutant (edited with RNP). B. Filter paper activities of Rut‐C30 and its mutants induced with 2% (w/v) wheat bran and 3% (w/v) Avicel. C. Filter paper activities of Rut‐C30 and its mutants induced with 1% (w/v) lactose. Scale bar = 1 cm. Error bars show the standard deviation of three replicates. All *P* values are obtained from two‐tailed *t* tests using Microsoft Excel: *P < 0.05, **P < 0.01, ***P < 0.001.

To ensure that transient introduction of RNPs would not disrupt the expression and function of other genes, the morphology and important phenotypic traits, such as growth and cellulase activity of the selected *T. reesei* transformants with *Trura5* mutagenesis were compared with those of their parental strains as well as uridine‐dependent mutants generated through ultraviolet mutagenesis or by using the Cas9 plasmid and gRNA (Liu *et al*., 2015). The mycelial growth and sporulation of six uridine‐dependent mutants were observed on potato dextrose agar (PDA) medium with or without uridine after 4 days of incubation. These included three randomly selected RNP‐directed mutants (3x‐1, 3x‐2 and 3x‐3; Fig. 1), an ultraviolet‐directed mutant derived from Rut‐C30 (1D4‐6, harbouring a 5 bp insertion in the open reading frame of *Trura5*), a mutant selected by transforming *Trura5*‐targeted gRNA into a Cas9 expressed chassis under the control of the inducing promoter Pcbh1 (AR3‐5; Liu *et al*., 2015) and a mutant selected by transforming *Trura5*‐targeted gRNA into a Cas9 expressed under the control of the constitutive promoter Ppdc (UPDC; Liu *et al*., 2015). On PDA plates without uridine, only the prototrophic strain Rut‐C30 formed spores; six mutants showed defects in mycelial growth or sporulation (Fig. 1A). By contrast, the growth and sporulation of the mutants on PDA plates containing uridine were not affected (Fig. 1A). Filter paper activities (FPAs) were measured using the filtrates from cultures of these mutants and their parental strains induced with different media including those containing 1% (w/v) lactose or 3% (w/v) Avicel microcrystalline cellulose plus 2% (w/v) wheat bran. No significant differences in FPA were observed among the RNP‐directed mutants 3x‐1, 3x‐2 and 3x‐3, the ultraviolet‐directed mutant 1D4‐6 and the parental strain Rut‐C30 in MM with wheat bran plus Avicel (Fig. 1B) or with lactose (Fig. 1C) as inducers. However, the FPAs of mutants based on Cas9 plasmids and gRNA were affected. FPA of the mutant AR3‐5 decreased by more than 50% FPA (*P* < 0.01) when Avicel plus wheat bran were used as inducers (Fig. 1B), while its FPA was not affected under induction by lactose (Fig. 1C). By contrast, the mutant UPDC lost 7.2% (*P* < 0.05) FPase activity in lactose medium, but its FPase activity was unaffected in medium containing Avicel plus wheat bran, compared with Rut‐C30. This suggested that *in vivo* expression of Cas9 might impact some ordinary physiological and biochemical processes in the transformants, which could be related to the endogenous promoter employed to express Cas9 protein. This speculation was supported by the relative expression levels of the endogenous genes *cbh1* and *pdc* corresponding to the promoters Pcbh1 and Ppdc, measured by quantitative real‐time PCR (Fig. S2).

### RNP‐directed homologous recombination in *T. reesei*

The CRISPR/Cas9 system based on RNPs is also conducive to homologous recombination between endogenous and exogenous DNA. We used *poura5* [an exogenous URA5 gene, encoding URA5 in *Penicillium oxalicum* (Liu *et al*., 2013)] as a selectable marker and the endogenous putative methyltransferase Lae1‐encoding gene (Trire2|41617, revised as NCBI accession no. AFX86442.1; Seiboth *et al*., 2012) as a target to evaluate if and to what extent RNPs support homologous recombination events. The uridine auxotrophic mutant 3x–1 (derived from Rut‐C30) was used as the parental strain. RNPs (170 nM RNP‐*Trlae1*, Cas9 assembled with gRNA_*Trlae1*, template: gRNA_M_*Trlae1*; Table S1) and 20 nM donor DNA (dDNA‐*lae1*‐1k, containing the Pgpda‐*poura5*‐Ttrpc cassette and the 1.0 kb 3ʹ and 5ʹ flanking regions of *lae1* obtained by overlapping PCR; Fig. S3; Table S1) were co‐transformed into *T. reesei* protoplasts using the optimized procedure with Triton X‐100 (Table 1). The transformants could be easily selected in minimal medium after 3 days of incubation at 28°C. Based on diagnostic PCR, only one of the nine selected transformants showed the correct homologous recombination at the target site (Fig. S3A). This result might be due to the dominance of NHEJ when the protoplasts were in inappropriate phases of the cell cycle. Because HDR (homology‐directed repair) is restricted to the late S and G2 phases rather than the G1 phase, we attempted to further prolong the incubation time at 20°C (from 25 to 50 min) to reduce the occurrence of NHEJ. Consequently, all 21 transformants showed significantly weakened sporulation and pigmentogenesis, consistent with the morphology and phenotype of the Δ*lae1* strain (Liu *et al*., 2013; Fig. S4). Further sequencing analyses of the selected transformants demonstrated that all mutants had undergone the correct homologous recombination process at the cleavage site (Fig. S3B).

The effects of the length of homology arms on recombination frequency were also investigated. The uridine‐dependent mutant 3x–1 was transformed with RNP‐*lae1* and a set of donor DNAs with homology arms of various lengths: 0.02 kb, 0.05 kb and 0.2 kb (dDNA*lae1–*0.02k, dDNA*lae1–*0.05k and dDNA*lae1–*0.2k respectively; Table S1). After co‐transformation with 170 nM RNPs and 20 nM donor DNAs using the optimized procedure with Triton X‐100 and further prolonging the incubation time to 50 min, all transformants thus obtained showed the same morphology and phenotype as the Δ*lae1* strain, indicating the loss of Lae1 function (Fig. S4). Twenty‐three random transformants from each transformation were selected for further verification through diagnostic PCR (Fig. S3). The homologous recombination frequencies with 0.02, 0.05 and 0.2 kb homology arms were 56.5% (Fig. S3C), 73.9% (Fig. S3D) and 100% (Fig. S3E), respectively. The results demonstrated that a pair of 20 bp homology arms was sufficient to routinely achieve efficient homologous recombination integration using the CRISPR/Cas9 system in *T*. *reesei*. After amplifying and sequencing the flanking sequences of the target sites, we showed that those transformants that could not successfully undergo homologous recombination had lost their homology arms in the flanking sequences (Fig. S3). This suggests that the probability of NHEJ repair may increase when the homology arms are too short (Table 2); however, it could be reduced by prolonging the incubation time of the transformation procedure. The control transformations without RNPs only resulted in one positive colony (Fig. S4F–I) with the right HDR at the *Trlae1* locus (Fig. S3F–I) by loading donor DNA with 1.0 kb homology arms. This indicates that RNPs improve the frequency of homologous recombination.

**Table 2:** RNP‐directed homologous recombination frequencies using homology arms of various lengths.

Homology arm size (kb)	No. of analysed transformants	Loss *lae1* function	HDR frequency (%)	NHEJ frequency (%)
1	21	21 (100%)	100.00	0.00
0.2	23	23 (100%)	100.00	0.00
0.05	23	23 (100%)	73.91	26.09
0.02	23	23 (100%)	56.52	43.48

### DNA‐free gene disruption using RNPs in *T. reesei*

**Fig. 2:**
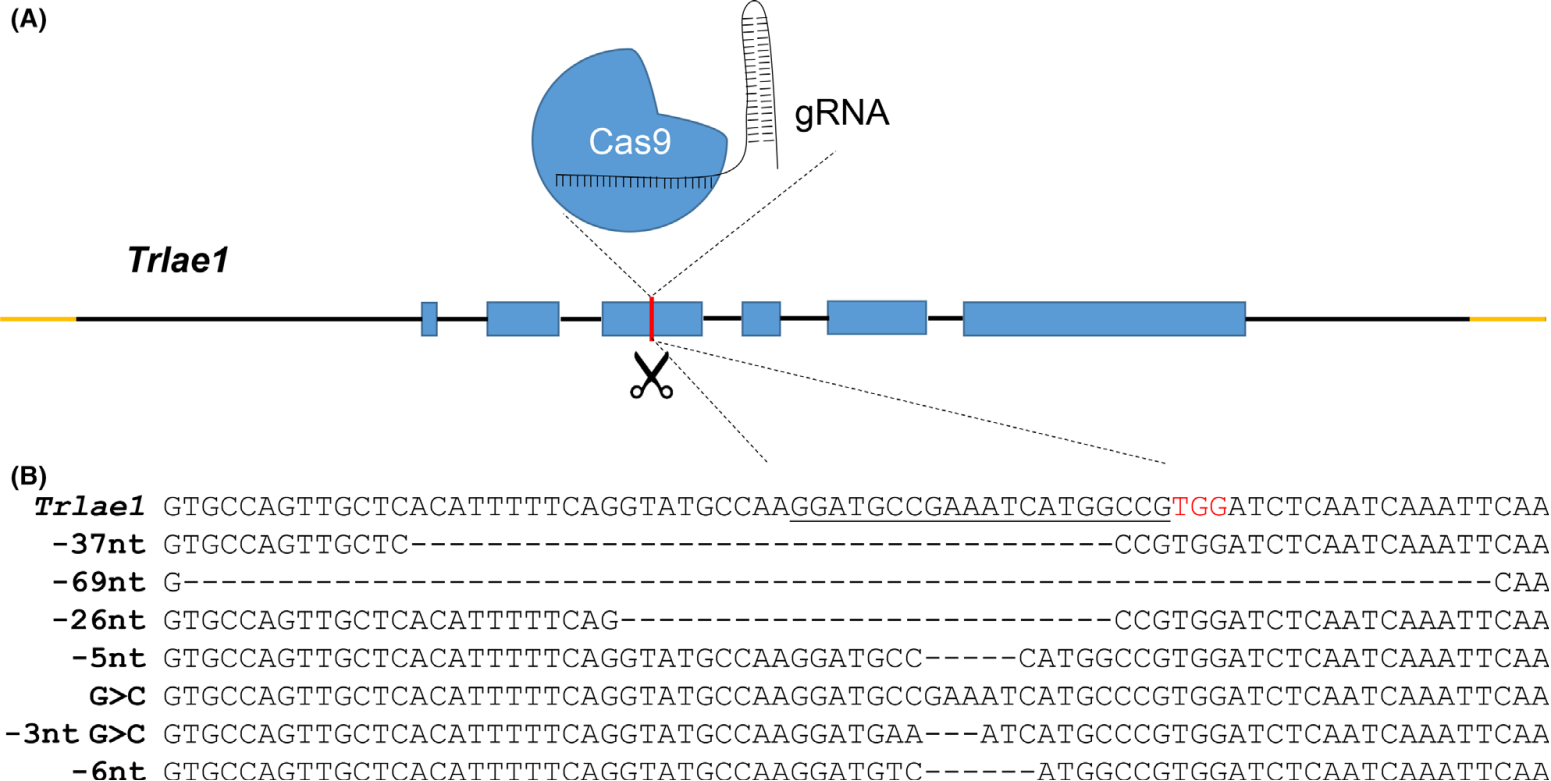
*Trlae1* disruption based on RNP transformation without DNA fragment or selectable marker addition. A. Schematic representation of *Trlae1* gene disruption through the RNP‐based CRISPR/Cas9 strategy. B. Sequence verification of the *lae1* gene from white colonies. The protospacer sequence is underlined and the PAM sequence is shown in red. ‘‐’ indicates deletion and ‘>’ indicates substitution mutation.

The RNP approach was found to allow straightforward and efficient genome editing in *T*. *reesei*, which could be compared with the *in vivo* expression level of the CRISPR‐Cas9 system. On the other hand, it is possible to edit a genome using RNP without adding donor DNA. Therefore, the frequency of gene disruption using only RNPs was tested. Diluted protoplasts (1 × 10^4^) were transformed with RNP‐*lae1* (170 nM RNP) and transferred to regeneration medium for sporulation (diluted to about 1000 protoplasts per plate). On regeneration medium, 95 colonies were randomly selected and transferred to four 24‐well plates along with the parental strain, from which seven colonies (7.37%) showed white phenotypes after 7 days of growth (Fig. S5). For further verification, a pair of primers flanking the *Trlae1* gene was designed and used for PCR amplification and sequencing of DNA fragments from the gDNA of those seven transformants. Mutations were observed in *Trlae1* of all seven mutants (Fig. 2), and the DNA‐free gene disruption strategy using RNPs successfully disrupted the function of TrLae1. This finding indicates that we could obtain 1–2 target mutants without addition of any exogenous DNA from 30 randomly selected transformants. Elimination of transgenic integration, DNA insertion and marker selection in the generated mutants is highly desirable for public acceptance of genome‐edited organisms (Kennedy *et al*., 2009; Kanchiswamy, 2016).

### The RNP CRISPR/Cas9 system for other filamentous fungi

To verify the versatility of the optimized *in vitro* CRISPR/Cas9‐guided genome editing system, we also carried out RNP transformation using the optimized PEG‐mediated procedure in the entomogenous fungus *Cordyceps militaris* and the industrial fungus *Aspergillus oryzae*. In *C*. *militaris*, gRNA (gRNA_*Cmura5*, template: gRNA_M_*Cmura5*) was designed to target *Cmura5* (Cormi1|4327; Table S1). When loaded with 170 nM RNPs, all (100%) 12 randomly selected *C*. *militaris* transformants had mutations in the vicinity of the cleavage site of *Cmura5* using the optimized transformation procedure with Triton X‐100 (Fig. S6). In *A*. *oryzae*, we targeted *AopyrG* (*Aoura3* in *A*. *oryzae*; AO090011000868; Table S1), encoding orotidine‐5ʹ‐phosphate decarboxylase. However, obtaining transformants from 5‐FOA‐containing plates after transformation of RNPs (gRNA_*AopyrG*, template: gRNA_M_*AopyrG*) into *A*. *oryzae* was difficult when using the same procedures for *T*. *reesei* and *C*. *militaris*. Only three *A*. *oryzae* transformants were obtained after three rounds of transformation. None of these three transformants could grow when transferred to a new screening plate containing 5‐FOA. Because *A*. *oryzae* spores and protoplasts usually contain multiple nuclei (Maruyama *et al*., 2001; Katayama *et al*., 2019), endocytic RNPs may be insufficient for genomic editing of all nuclei in protoplasts. In addition, these multinucleate cells showed superior germination and growth efficiency compared with cells with a single nucleus (Ishi *et al*., 2005). This difference might explain the difficulty of obtaining homozygous transformants from *A*. *oryzae* through simply increasing the penetration of RNPs.

A more versatile and convenient technique for genome editing is required for efficient molecular breeding of engineered fungal strains with multinucleate protoplasts. *A*. *oryzae* mycelia were pre‐treated with inositol (100 mg l^−1^) or benomyl (1 mg l^−1^) and then collected for protoplast generation. Gene *wA* (AO090102000545; gRNA_*AowA*, template: gRNA_M_*AowA*; Table S1), encoding a polyketide synthase required for conidial pigmentation in *A*. *oryzae*, was selected as the deletion target using donor DNA (primers: argB‐wA‐F and argB‐wA‐R) containing the 37 or 40 bp flanking sequences of *wA* as homological arms, with a wild‐type *argB* selection marker replacing the target gene via HDR (Table S1). RNPs (170 nM) and donor DNA (20 nM) were transformed into *A. oryzae* protoplasts (˜2 × 10^5^ protoplasts) via PEG‐mediated transformation facilitated by Triton X‐100. The resulting number of CFUs was calculated and the targeting efficiency was analysed through PCR analyses (primers: ΔwA‐F and ΔwA‐R) after 3 days of growth on Czapek–Dox (CD) medium containing 1.5 g l^−1^ methionine and 0.1 g l^−1^ adenine (Fig. S7). The correct insertion of donor DNA was verified based on the PCR products obtained from the 5′ and 3′ breakpoint junctions (Fig. S7). Transformation using 100 mg l^−1^ inositol resulted in 40.0% Δ*wA* homokaryotic transformants, while that using 1 mg l^−1^ benomyl resulted in 71.4% Δ*wA* homokaryotic transformants with the correct donor DNA insertion at the target site. When the transformation was performed without inositol or benomyl, no Δ*wA* homokaryotic transformant was obtained, although 80% (12/15) of the selected colonies had the Δ*wA* karyotype mixed with the wild karyotype (Table 3; Fig. S7). In the control transformation, performed without RNPs, none of the 12 transformants were observed to undergo HDR at the *wA* locus (Fig. S7E). We also tested the editing efficiency of *AopyrG* by transforming 170 nM gRNA_*AopyrG* companied by 20 nM donor DNA (with 0.8 and 0.9 kb flanking sequences) into *A. oryzae* protoplasts (˜2 × 10^5^ protoplasts; Fig. S8). The optimized procedure using benomyl led to 59.1% (13 of 22) of transformants with the Δ*AopyrG* karyotype and 54.5% (12 of 22) with homokaryotic Δ*AopyrG* obtained via HDR, among randomly selected transformants on selective media containing 5‐FOA (Fig. S8A). Of the 22 transformants, three were obtained by the NHEJ pathway (Fig. S8D). In total, 72.73% of transformants were homokaryotic. In the control transformation without RNPs, only one transformant was obtained, although HDR was detected at its *AopyrG* locus (Fig. S8C). This suggests that the additional RNP boosts the efficiency of HDR by more than 10‐fold when the same transformation procedure is used.

**Table 3:** Percentage of RNP‐directed homokaryotic transformants using different chemical reagents

Chemical reagents	No. of analysed CFU	Homokaryotic Δ*wA*	CFUs with Δ*wA* karyotype
None	15	0 (0.00%)	12 (80.00%)
100 mg l^−1^ inositol	15	6 (40.00%)	13 (86.67%)
1 mg l^−1^ benomyl	21	15 (71.43%)	18 (85.71%)

### RNP‐stimulated multiplexed homologous recombination in filamentous fungi

As a robust expression host for secretory proteins, a genetic approach that could target multiple genes would greatly benefit strain engineering. To test whether the optimized RNP transformation procedure could be applied to simultaneous multi‐target editing in filamentous fungi, we used a gene (ACLA_76850) encoding sesterterpene cyclase (AcOS catalyses geranylfarnesyl diphosphate to produce sesterterpene ophiobolin F; Chiba *et al*., 2013) from *Aspergillus clavatus* to replace *cbh1* (Trire2_123989, gRNA_*Trcbh1*, template: gRNA_M_*Trcbh1*), *cbh2* (Trire2_72567, gRNA_*Trcbh2*, template: gRNA_M_*Trcbh2*) and *eg1* (Trire2_122081, gRNA_*Treg1*, template: gRNA_M_*Treg1*) in *T*. *reesei* (Table S1). The donor DNA contained flanking sequences of 500 to 1000 bp as homological arms (Fig. S9A–D) and the *ura5* selection marker to replace the entire open reading frame of each target gene via HDR (Fig. S9). Two of 20 transformants (10.0%) showed insertion of the correct donor DNA at all three genomic targets. Eight of the transformants (40.0%) contained two genome‐edited sites. The rest of the transformants (50.0%) showed replacement of the *Trcbh1* gene (Fig. S9A–D). In the control transformation performed without RNPs, three of 20 transformants (15.0%) were exhibited replacement of the *Trcbh1* gene (Fig. S9E). Two transformants (10.0%; Fig. S9F) at the *Trcbh2* locus (5.0%) and one at the *Treg1* locus (Fig. S9G) exhibited HDR. None of these were edited at multiple sites.

We also used the gene encoding cyclase AcOS to replace the target genes *amyA* (AO090003001591), *amyB* (AO090120000196) and *amyC* (AO090023000944; all three genes sharing the same spacer sequence, gRNA_*Aoamy*, template: gRNA_M_*Aoamy*) in *A*. *oryzae* by the optimized transformation procedure using 1 mg ml^−1^ benomyl (Fig. S10; Table S1). Donor DNA containing 1423 and 529 bp flanking sequences as homological arms and the wild‐type *adeA* selection marker was used to replace the entire open reading frame of each of the three target genes via HDR (Table S1; Nemoto *et al*., 2012). Nine transformants were screened after 3 days of growth on Czapek–Dox medium containing 1.5 g l^−1^ methionine and 1.5 g l^−1^ arginine. Two of these selected transformants (22.2%) had the correct donor DNA insertion at all three genomic targets. Five (55.6%) exhibited editing of two target genes (Fig. S10). This indicates that RNP‐based multiplexing manipulation could be successful in fungi with multinucleate protoplasts. In the control transformation performed without RNPs, one of 10 transformants exhibited the Δ*AoamyB* and Δ*AoamyC* karyotypes (Fig. S10E and F). Three of the transformants exhibited HDR at the Δ*AoamyB* locus (Fig. S10E). However, all of these transformants were mixed with those with the wild‐type karyotype in the control transformation (Fig. S10).

## Discussion

In this study, the efficiency of a CRISPR/Cas9 RNP‐mediated genome editing system was significantly improved by using the chemical reagent Triton X‐100. Surfactants are widely used to solubilize, separate and extract biological membrane components, particularly integral proteins (Jones, 1999; Seddon *et al*., 2004). Previous research has shown that a low dosage of Triton X‐100 induces membrane permeabilization under sub‐solubilization conditions (Ahyayauch *et al*., 2012; Mattei *et al*., 2017). In this study, adding Triton X‐100 dramatically increased the CFU numbers of RNP transformations, which could be attributed to greatly improved efficiency of RNP penetration. Other surfactants, such as Tween 80 and NP40, may also be able to increase RNP penetration into cells.

**Fig. 3:**
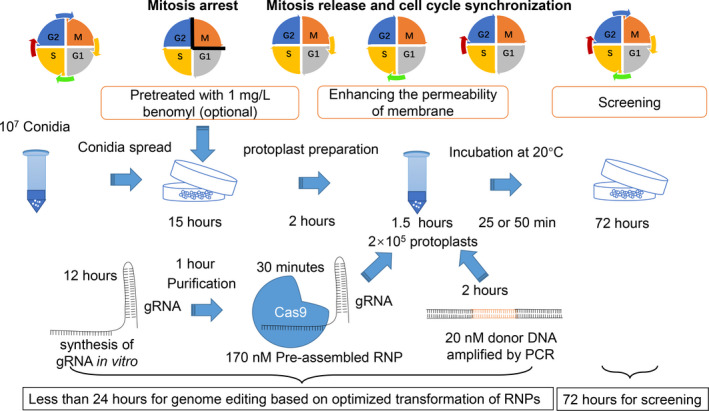
Overview of RNP‐assisted genome editing in filamentous fungi. Downscaling of the final optimized transformation procedure. Mature conidia were collected with 0.02% Tween 80. The germinated conidia are treated with 1 mg l^−1^ benomyl on PDA plates to induce mitotic arrest (required for fungi with multinucleate protoplasts). After 15 h, mycelia were washed with sterilized water three times to remove benomyl. Protoplast preparation followed a previously reported procedure, and the protoplast concentration was diluted to 10^6^ ml^‐1^. RNP(s) and donor DNA(s) (optional) were added to 200 μl protoplast suspensions. After addition of PEG 6000 solution, the surfactant Triton X‐100 is incorporated at a final concentration of 0.006% (w/v). The incubation time was prolonged to more than 25 min for full release of mitotic arrest (25 min for NHEJ, 50 min for HDR). Protoplast regeneration and transformant screening also followed a previously reported procedure. M: mitosis phase, G1: G1 phase in interphase, S: S phase in interphase, G2: G2 phase in interphase.

In *T*. *reesei* or *C*. *militaris*, filamentous fungi that do not form multinucleate spores, addition of the chemical reagent Triton X‐100 was sufficient for routine genome editing. However, in *A*. *oryzae*, a fungus that is prone to form multinucleate spores and protoplasts, isolation of pure transformants from transient transformation with RNPs is extremely difficult. The addition of inositol or benomyl increases the formation of monokaryotic protoplasts, which can greatly improve the efficiency of obtaining homozygous transformants and eliminate the multi‐round single‐spore isolation process in the late stage of transformation. Therefore, these reagents greatly shorten the time required for genome editing. Inositol, or more precisely *myo*‐inositol, plays an important role as the structural basis for a number of secondary messengers in eukaryotic cells, known as inositol phosphates (Thomas *et al*., 2016). Pyrophosphates (IPPs) are essential for chromosome transmission fidelity, and chromosome transmission fidelity was enhanced with increased cellular IPP levels (Kwon *et al*., 2019). The addition of inositol led to an increase in the concentration of intracellular IPPs, which in turn led to increased ratios of monokaryotic cells during cell division (Kwon *et al*., 2019). On the other hand, it has been reported that over half of the major cell wall proteins are modified by the addition of a glycosylphosphatidylinositol anchor in fungi (Free, 2013). All glycosylphosphatidylinositol anchors have a core structure consisting of a phosphatidylinositol, which is attached to an oligosaccharide chain terminated by a phosphoethanolamine. The oligosaccharide consists of a glucosamine residue (initially added as GlcNAC and then deacetylated) attached to the inositol by α‐1,6‐linkage, followed by three mannose residues (De Groot *et al*., 2003). Inositol promotes the formation of fungal cell walls, which may also be involved in a proportion of monokaryotic cells. Compared with inositol, benomyl confers a much greater benefit to RNP‐based genome editing in *A*. *oryzae*. By treating the cell with 1 mg l^−1^ benomyl, cell nucleus haploidization was induced in the stable diploid strain. This method has been confirmed to be reliable for obtaining haploid strains in *A*. *oryzae* (Hara *et al*., 2012). In addition to increasing the proportion of monokaryotic cells, pretreatment of mycelium with benomyl caused mitotic arrest and cell cycle synchronization (Kiso *et al*., 2004; Zou *et al*., 2006). In the process of transformation, due to the removal of benomyl, cells entered the G1 phase, S phase and then G2 phase synchronously. In synchronized cells, RNP‐mediated HDR was increased dramatically relative to experiments in unsynchronized cells (Lin *et al*., 2014). With the help of chemical reagents, the final optimized protocol can be entirely performed in *ca*. 4 days (Fig. 3).

In summary, we developed an efficient CRISPR/Cas9 RNP‐mediated genome editing system for filamentous fungi. This system can delete genes using short homology arms or disrupt genes without the introduction of foreign DNA, including selection markers. Moreover, our system can be applied in multi‐target homologous recombination in fungi with mononucleate or multinucleate protoplasts. The transient introduction of RNPs may prevent the side‐effects caused by transgenic procedures and minimize the risk to host cells posed by the*in vivo* expression of a heterologous Cas9 endonuclease. Such improvements might facilitate the establishment of more efficient transgene‐independent genome editing procedures in fungi.

## Experimental procedures

### Strains and culture media

The *T. reesei* strain Rut‐C30 (56765) was purchased from American Type Culture Collection (ATCC, Manassas, VA, USA). *Aspergillus oryzae* NSAR1 (*niaD* ^−^, *sC* ^−^, Δ*argB*, *adeA* ^−^; kindly provided by Dr. Dan Hu from Jinan University) and *C. militaris* CM01 also served as the host for RNP to test editing efficiency. *T. reesei* and *C. militaris* were grown on potato dextrose agar medium (200 g potato infusion, 20 g dextrose, 20 g agar to 1 l water). The minimal medium for *T. reesei* contained 4.0 g l^−1^ KH_2_PO_4_, 2.8 g l^−1^ (NH_4_)_2_SO_4_, 0.6 g l^−1^ MgSO_4_•7H 2 O, 0.5 g l^−1^ CaCl_2_, 0.6 g l^−1^ urea, 3.0 g l^−1^ tryptone, 1.0 g l^−1^ Tween 80, 5 g l^−1^ CaCO_3_, 0.01 g l^−1^ FeSO_4_•7H_2_O, 0.0032 g l^−1^ MnSO_4_•H_2_O, 0.0028 g l^−1^ ZnSO_4_•7H_2_O and 0.004 g l^−1^ CoCl_2_ and was adjusted to pH 5.5. Protoplast regeneration medium for *T. reesei* and *C. militaris* contained 182.17 g l^−1^ sorbitol, 1.0 g l^−1^ MgSO_4_•7H_2_O, 1 g l^−1^ KH_2_PO_4_, 0.6 g l^−1^ (NH_4_)_2_SO_4_ 0.3 g l^−1^ tri‐sodium citrate•2H_2_O, 10.0 g l^−1^ glucose•H_2_O, 0.005 g l^−1^ FeSO_4_•7H_2_O, 0.0016 g l^−1^ MnSO_4_•H_2_O, 0.0014 g l^−1^ ZnSO_4_•7H_2_O, 0.0012 g l^−1^ CoCl_2_ and 12 g l^−1^ agar. *A. oryzae* was grown on CD medium [4.66 g l^−1^ (NH_4_)_2_SO_4_, 0.52 g l^−1^ KCl, 1.52 g l^−1^ K_2_HPO_4_, 0.49 g l^−1^ MgSO_4_•7H_2_O, 10.0 g l^−1^ glucose, 0.001 g l^−1^ FeSO_4_•7H_2_O, 0.0088 g l^−1^ ZnSO_4_•7H_2_O, 0.0004 g l^−1^ CuSO_4_•5H_2_O, 0.0001 g l^−1^ Na_2_B_4_O_7_•10H_2_O, 0.00005 g l^−1^ (NH_4_)_6_Mo_7_O_24_•4H_2_O, 1.5 g l^−1^ of methionine, 1.5 g l^−1^ arginine, 0.1 g l^−1^ adenine, 9.0 g l^−1^ of (NH_4_)_2_SO_4_ and 15.0 g l^−1^ of agar, pH 5.5]. Protoplast regeneration CD medium for *A. oryzae* contained 46.8 g l^−1^ NaCl, 0.52 g l^−1^ KCl, 1.52 g l^−1^ K_2_HPO_4_, 0.49 g l^−1^ MgSO_4_•7H_2_O, 10.0 g l^−1^ glucose, 0.001 g l^−1^ FeSO_4_•7H_2_O, 0.0088 g l^−1^ ZnSO_4_•7H_2_O, 0.0004 g l^−1^ CuSO_4_•5H_2_O, 0.0001 g l^−1^ Na_2_B_4_O_7_•10H_2_O, 0.00005 g l^−1^ (NH_4_)_6_Mo_7_O_24_•4H_2_O, 12.0 g l^−1^ agarose (pH = 6.5) supplemented with appropriate nutrients [4.66 g l^−1^ of (NH_4_)_2_SO_4_ for *niaD* ^−^ genotype, 1.5 g l^−1^ of methionine for *sC* ^−^ genotype, 1.5 g l^−1^ arginine for Δ*argB* genotype, 0.1 g l^−1^ adenine for *adeA* ^–^ genotype, 2.0 g l^−1^ uracil and 4.88 g l^−1^ uridine for Δ*pyrG* genotype]. Uracil‐requiring mutants were selected on media containing 5‐fluoro‐orotic acid (1.5 g l^−1^ for *T. reesei*, 0.8 g l^−1^ for *C militaris* and 2.0 g l^−1^ for *A. oryzae*).

### Cas9 protein and guide RNAs

Cas9 protein tagged with a nuclear localization signal was purchased from Novoprotein, Inc. (Shanghai, China). Templates (Table S1) for guide RNA transcription were generated by oligo‐extension using I5 polymerase (Tsingke, Beijing, China). Target sequences were designed *in silico* using Cas‐Designer and Cas‐OFFinder (http://www.rgenome.net/cas‐offinder/) to reduce off‐target. For targeting sequence information, see Table S1. Guide RNAs were *in vitro* transcribed through runoff reactions using the T7 RNA polymerase (New England BioLabs, Ipswich, MA, USA) according to the manufacturer's protocol. Mix 1 μl of template DNA, ATP solution, CTP solution, GTP solution, UTP solution, 10 × reaction buffer (0.4 M Tris‐HCl, 60.0 mM MgCl_2_, 10.0 mM dithiothreitol, 20.0 mM spermidine, pH 7.9) and enzyme mix. Add water to make up a total volume of 10 μl. Gently flick the tube or pipette the mixture up and down gently, and then microfuge tube briefly to collect the reaction mixture at the bottom of the tube. Incubate overnight at 37°C. The reaction mixture was treated with DNase I (New England BioLabs) in 1 × DNase I reaction buffer (10.0 mM Tris‐HCl, 2.5 mM MgCl_2_, 0.5 mM CaCl_2_, pH 7.6). Transcribed gRNAs were resolved on DEPC‐treated water for quality control. The gRNA fragments were pre‐assembled with Cas9 to form RNPs using Cas9 Kit (Novoprotein, Shanghai, China). Prior to the transformation into fungal protoplasts, RNP complexes were assembled in 20 μl volume reaction containing 6 μl CRISPR nuclease (1.875 μM), 2 μl 10 × Cas9 activity buffer (0.2 M HEPES, 1.5 M KCl, 5.0 mM dithiothreitol, 1.0 mM EDTA, 0.1 M MgCl_2_, pH 7.5), 5 μl gRNA (˜ 2 μM) and 7 μl of nuclease‐free water. The mixture was incubated at 37°C for 15 min to allow RNP complex formation. For multiplex targeting, each target RNP complex was formed separately.

### Protoplast transformation of RNP

Protoplast transformation of RNPs was carried out using a modified polyethylene glycol‐mediated protoplast transformation procedure (Liu *et al*., 2015). Before protoplast preparation, 100 mg l^−1^ inositol or 1 mg l^−1^ benomyl (to form monokaryotic protoplasts and synchronize cell cycles; extra step to form monokaryotic protoplasts) treated mycelia for 15 h and then washed by sterilized water three times to remove inositol and benomyl. Protoplasting was performed using 0.5% lysing enzymes from *Trichoderma harzianum* (Sigma‐Aldrich, St. Louis, MO, USA) in solution I (1.2 M sorbitol and 0.1 M KH_2_PO_4_, pH 5.5) at 28°C (*T. reesei* and *C. militaris*) or 30°C (*A. oryzae*) for 2 h. Protoplasts were filtered by Miracloth (Sigma‐Aldrich) and centrifuged at 800 *g* for 10 min at 4°C, washed with Solution I and centrifuged at 800 *g* for 10 min at 4°C. Then, protoplasts were adjusted to about 10^6^ cells ml^−1^ by adding Solution 2 (1 M sorbitol, 50 mM CaCl_2_, 10 mM Tris‐HCl, pH = 7.5) and Solution 3 [25% (w/v) of PEG6000, 50 mM CaCl_2_, 10 mM Tris‐HCl, pH = 7.5] in 4/1 volume ratio. To the protoplast solution (200 μl) was added different amount of RNPs. Triton X‐100 [0.006% (w/v) final concentration in transformation reaction] was used to assist RNP conveying across cell membrane. The aliquot was incubated on ice for 20 min and then Solution 3 (3 ml) added to the aliquot. After 25 min of incubation (optimized from 5 to 25 min) at 20°C, Solution 2 (8 ml) added to the mixtures. The mixtures were poured into 50 ml melted regeneration medium (cooled to 50°C) and divided into three plates. For selection‐free gene disruption stimulated by RNP, the protoplasts were diluted to 10^3^ for each transformation. RNP‐transformed protoplasts were re‐suspended in 1 ml transformation reaction with 0.006% (w/v) Triton X‐100 and then spread on PDA containing 1 M sorbitol.

In co‐transformation of RNPs with donor DNAs, the procedure was almost the same. But the incubation with RNPs and donor DNAs was prolonged from 25 to 50 min at 20°C. The donor DNAs for *T. reesei lae1* HDR were containing the 5ʹ and 3ʹ flanking sequences of *lae1* and the selectable marker cassette (the *ura5* gene from *P. oxalicum* controlled by the Pgpda promoter and Ttrpc terminator, Pgpda‐poura5‐Ttrpc; Fig. S3; Table S1) was generated by overlapping PCR using I5 (Tsingke, Beijing, China). Besides the 1 kb flanking sequences (dDNA‐lae1), the 0.2, 0.05 and 0.02 flanking sequences of *lae1* were fused to the selectable marker (Fig. S3; Table S1). For testing multiple genome editing in *T. reesei*, *AcOS* gene (ACLA_76850; from *Aspergillus clavatus)* produced a sesterterpene alcohol, ophiobolin F and three minor sesterterpene hydrocarbons (Chiba *et al*., 2013) was selected to target *T. reesei cbh1, cbh2* and *eg1* (coding the main secreted proteins occupying more than 80% total secreted proteins in enzyme preparation of *T. reesei*) using donor DNAs containing the 500˜1000 bp flanking sequences and *ura5* selection marker (Fig. S9; Table S1). To test multiplexed genome editing in *A. oryzae*, we also used *AcOS* gene to replace the *amyA, amyB* and *amyC* of *A. oryzae*. Donor DNAs containing 1423 and 529 bp flanking sequences, *AcOS* expression cassette and wild *adeA* expression cassette (selection marker) were used to replace the whole open reading frames of three targeted genes (Fig. S10; Table S1).

All the sequence analyses of transformants were carried out by BioSune Biotechnology (Shanghai, China).

### Enzyme activity assays

*T. reesei* and its mutants were spread on PDA plates and were grown at 28°C for about 7 days and then stored at 4°C after conidia formed. The conidia of the fungal transformants were collected from PDA plates and inoculated into 50 ml flasks containing 10 ml Sabouraud dextrose broth (SDB) and cultured for 2 days at 28°C and 200 rpm on a rotary shaker. Subsequently, 1 ml of the culture was transferred to flasks with 10 ml minimal medium plus different inducers [3% (w/v) cellulose powder (CF‐11, Whatman, Maidstone, England) and 2% (w/v) wheat bran (ground to less than 0.5 mm in diameter by a mill at the lab) as inducer or 1% (w/v) lactose as inducer] and incubated at 28°C and 200 rpm.

The crude secreted enzymes of fungal strains (the culture filtrate of *T. reesei* or its transformants was collected by a centrifugation at 4°C and 8000 *g* for 10 min after being cultured for 7 days). FPA was assayed by measuring the amount of reducing sugar released from filter paper (Whatman, Maidstone, England) using the DNS method (Liu *et al*., 2015). The assay mixture contained a specific amount of diluted enzyme, 0.1 g filter paper and 80 μl 50 mM saline sodium citrate buffer (pH 5.0). The mixture was incubated at 60°C for 10 min; 120 μl DNS was added to stop the reaction, followed by incubation for 5 min in boiling water. Photometric assays were analysed at OD 540 using a Varioskan Flash microplate reader (Thermo Scientific, Rockford, IL, USA). All the enzyme assays were performed for three times.

## Conflict of interest

The authors declare that they have no conflict of interest.

## Supporting information


**Fig. S1.** Verifying *Trura5* mutation after RNP‐based gene disruption. Chromatogram and alignment of *Trura5* sequences of *T. reesei* and its mutants obtained by transformation with 60 nM RNPs (A), 100 nM RNPs (B), 170 nM RNPs (C) or 60 nM RNPs and facilitated using 0.006% Triton X‐100 (D), 100 nM RNPs loaded and facilitated using 0.006% Triton X‐100 (E), 170 nM RNPs loaded and facilitated using 0.006% Triton X‐100 (F). WT: wild type strain Rut‐C30. 1‐1, 2‐1, 2‐2, and 3‐1˜3‐6: mutants generated by directly transformation of RNPs. 1x‐1, 1x‐2, 2x‐1˜2x‐8 and 3x‐1˜3x‐12: mutants stimulated by RNP facilitated by 0.006% Triton X‐100.
**Fig. S2.** Relative expression levels of *Trcbh1* and *Trpdc* in Rut‐C30 and its deriving strain AR3‐5 and UPDC. (A) Relative expression levels of the endogenous *Trcbh1* in AR3‐5 harboring a Cas9 expressed under the control of the inducing promoter Pcbh1 when induced by 2% (w/v) wheat bran plus 3% (w/v) Avicel. (B) Relative expression levels of *Trcbh1* in AR3‐5 induced by 1% (w/v) lactose. In AR3‐5, *Trcbh1* was downregulated 24 h and 48 h after induction of wheat bran and Avicels. (C) Relative expression levels of the endogenous *Trpdc* in UPDC harboring a Cas9 expressed under the control of the constitutive promoter Ppdc when induced by 3% (w/v) wheat bran plus 2% (w/v) Avicel. (D) Relative expression levels of *Trpdc* in UPDC induced by 1% (w/v) lactose. In UPDC, *Trpdc* was upregulated at 6 h and downregulated at 24 h and 48 h following induction by lactose. *Trsar1* (Trire2|61470) was used as the reference gene. The primers used are listed in (Table S1). Error bars show the standard deviation of three replicates. All *P* values were generated from two‐tailed *t*‐tests using Microsoft Excel: *P < 0.05, **P < 0.01, ***P < 0.001.
**Fig. S3.** Verifying the *Trlae1* locus in the selected transformants obtained by co‐transformation of different flanking sequences and RNPs. (A) Transformants obtained using 1000 bp flanking sequences and 25 min incubation at 20°C. (B) Transformants obtained using 1000 bp flanking sequences. (C) Transformants obtained using 200 bp flanking sequences. (D) Transformants obtained using 50 bp flanking sequences. (E) Transformants obtained using 20 bp flanking sequences. (F–I) Control transformation performed using various flanking sequences without RNPs. All 16 transformants obtained using 1000 bp flanking sequences were verified by PCR (F). However, 10 randomly selected transformants obtained from each control transformation using 200 bp (G), 50 bp (H) or 20 bp (I) flanking sequences were verified, as none of these transformants formed white colonies (Figure S4).
**Fig. S4.** Morphology of selected transformants with co‐transformation of different flanking sequences and RNP directing *Trlae1* locus. The transformants in each panel correspond one by one to these in Figure S3. The numbers that start with lowercase letters represent independent transformants of each transformation. wt: parent strain 3x‐1.
**Fig. S5.** Morphology of *Trlae1* disruption based on RNP transformation without DNA fragment and selectable marker. Randomly selected transformants on 24‐well plates with PDA medium. Transformants lost *Trlae1* function were marked with red circles.
**Fig. S6.** Verifying*Cmura5* mutation after RNP‐based gene disruption when 170 nM RNP loaded and facilitated using 0.006% Trition X‐100. All 12 randomly selected transformants were detected NHEJ in the targeting sequence. WT: wild type *C. militaris* Cm01. Cm‐1˜Cm‐12: randomly selected mutants directed by the optimized transformation procedure facilitated using Triton X‐100.
**Fig. S7.** Verifying the*AowA* disruption in the selected transformants obtained by co‐transformation of RNPs and *AoargB* selectable marker. (A) Verification PCR result for transformation performed with Triton X‐100. (B) Transformation performed with Triton X‐100 and 100 mg/L inositol in protoplast preparation. (C) Transformation performed with Triton X‐100 and 1 mg/L benomyl in protoplast preparation. (D) Schematic diagram for the *AowA* disruption. (E) Transformants of control transformation performed as (C), but without RNP. The numbers preceded by lowercase letters represent independent transformants from each transformation. M: marker. NSAR1: parent strain in this assay. RIB40, initial parent strain of NSAR1. ‐: negative control. Transformants marked with red numbers were repaired by HDR. Homozygous transformants were underlined. Red arrows: primers used to amplify donor DNAs. Yellow arrows: primers used for verification. All primers are listed in Table S1.
**Fig. S8.** Verification and sequencing for the*AopyrG* disruption. Verification PCR (A) and schematic diagram (B) for the*AopyrG* disruption which was performed using 1 mg/L benomyl and was incubated for 50 min at 20°C. (C) Control transformation was performed without RNP. The numbers on the gel pictures represented different transformants from the optimized transformation. M: marker. NSAR1: parent strain. ‐: negative control. T: the transformant obtained in the control transformation. Transformants marked with red characters were repaired by HDR, while these marked with green characters were repaired by NHEJ. Homozygous transformants were underlined. Red arrows: primers used to amplify donor DNAs. Yellow arrows: primers used for verification. All the primers are listed in Table S1. (D) Sequencing for the*AopyrG* gene of transformants without detectable HDR. Transformants 1, 13, 17 were detected NHEJ in the targeting sequence. And there was no mutation observed in *AopyrG* gene of transformants 9, 11, 12.
**Fig. S9.** Verifying*AcOS* insertion targeting *Trcbh1*, *Trcbh2*, *Treg1* in the selected *T. reesei* transformants from co‐transformation of RNPs and *poura5* selectable marker. (A) ˜ (D): Verification PCR for transformation performed using the optimized procedure with Triton X‐100. Verifying HDR at the *Trcbh1* (A), *Trcbh2* (B), and *Treg1* (C) loci in *T. reesei* transformants. (D) Verifying *AcOS* gene in genome of *T. reesei* transformants. Two of 20 transformants (10.0%, transformants a8 and a19) had correct donor DNA insertion at all of the genomic targets. Eight of them (40.0%, transformants a6, a7, a13, a14, a15, a16, a17 and a18) were conducted two sites (*Trcbh1*, *Treg1*) genome edited. And all the rest transformants (50.0%, transformants a1, a2, a3, a4, a5, a9, a10, a11, a12 and a20) replaced the *Trcbh1* gene. (E) ˜ (H): Verification PCR for control transformation performed without RNPs. Three of 20 randomly selected transformants had HDR at *Trcbh1* locus (E). Two transformants had replaced *Trcbh2* gene (F). One transformant was conducted *Treg1* edited correctly (G). *AcOS* insertion could be detected in all transformants obtained by the control transformation (H). However, the HDR efficiency (5%˜15%) was very low without RNP. None had multiplexed homologous recombination at the targeting genomic targets.
**Fig. S10.** Verifying*AcOS* insertion targeting *AoamyA*, *AoamyB*, *AoamyC* in the selected *A. oryzae* transformants from co‐transformation of RNPs and *AoadeA* selectable marker. (A) ˜ (C) Verification PCR for transformation performed using the optimized procedure with Triton X‐100 and benomyl. Verifying HDR at *AoamyA* (A), *AoamyC* (B), and *AoamyC* (C) loci in *A. oryzae* transformants. Two of 9 transformants (22.2%, transformants a6 and a10) had correct donor DNA insertion at all of the genomic targets. Five of them (55.6%) were conducted two genome‐edited sites (transformants a1, a2 and a5 at *AoamyB* and *AoamyC*, transformants a3 and a4 at *AoamyA* and *AoamyB*). And all the rest transformants (22.2%, transformants a7 and a8) replaced the *AoamyA* gene by *AcOS*. Except transformants a3 and a5, the rest transformants were homozygous transformants. Since the same 3’ flanking sequence (> 9 kb) shared with *AoamyB* and *AoamyC*, PCR was performed using primers AdeA‐F/ΔamyB‐R (B) and AdeA‐F/ΔamyC‐R (C). (D) ˜ (F) Verification PCR for the control transformation performed without RNP. None of 10 transformants had HDR at *AoamyA* (D). Four transformants (c1, c2, c4 and c9) replaced *AoamyB* gene (E). One transformant was replaced *AoamyC* gene (F). Due to the same 3’ flanking sequence, PCR results were shown in the same gel picture verified using primers AdeA‐F/ΔamyB‐R (E) and AdeA‐F/ΔamyC‐R (F). One of them (transformant c2) exhibited the Δ*AoamyB* and Δ*AoamyC* karyotypes. However, none of them was homozygous transformant in the control transformation.
**Table S1.** Oligonucleotides and gene sequences used in this study.
**Table S2.** Predicted off‐target sites.
