# Supplementary material for: Efficient genome editing in filamentous fungi via an improved CRISPR‐Cas9 ribonucleoprotein method facilitated by chemical reagents
Source: Microb Biotechnol. 2020 Aug 25;14(6):2343–55. doi: 10.1111/1751-7915.13652 (PMC8601184; doi:10.1111/1751-7915.13652)
Supplement: Supplementary file 1 — Fig. S1. Verifying Trura5 mutation after RNP‐based gene disruption. Chromatogram and alignment of Trura5 sequences of T. reesei and its mutants obtained by transformation with 60 nM RNPs (A), 100 nM RNPs (B), 170 nM RNPs (C) or 60 nM RNPs and facilitated using 0.006% Triton X‐100 (D), 100 nM RNPs loaded and facilitated using 0.006% Triton X‐100 (E), 170 nM RNPs loaded and facilitated using 0.006% Triton X‐100 (F). WT: wild type strain Rut‐C30. 1‐1, 2‐1, 2‐2, and 3‐1˜3‐6: mutants generated by directly transformation of RNPs. 1x‐1, 1x‐2, 2x‐1˜2x‐8 and 3x‐1˜3x‐12: mutants stimulated by RNP facilitated by 0.006% Triton X‐100. Fig. S2. Relative expression levels of Trcbh1 and Trpdc in Rut‐C30 and its deriving strain AR3‐5 and UPDC. (A) Relative expression levels of the endogenous Trcbh1 in AR3‐5 harboring a Cas9 expressed under the control of the inducing promoter Pcbh1 when induced by 2% (w/v) wheat bran plus 3% (w/v) Avicel. (B) Relative expression levels of Trcbh1 in AR3‐5 induced by 1% (w/v) lactose. In AR3‐5, Trcbh1 was downregulated 24 h and 48 h after induction of wheat bran and Avicels. (C) Relative expression levels of the endogenous Trpdc in UPDC harboring a Cas9 expressed under the control of the constitutive promoter Ppdc when induced by 3% (w/v) wheat bran plus 2% (w/v) Avicel. (D) Relative expression levels of Trpdc in UPDC induced by 1% (w/v) lactose. In UPDC, Trpdc was upregulated at 6 h and downregulated at 24 h and 48 h following induction by lactose. Trsar1 (Trire2|61470) was used as the reference gene. The primers used are listed in (Table S1). Error bars show the standard deviation of three replicates. All P values were generated from two‐tailed t‐tests using Microsoft Excel: *P < 0.05, **P < 0.01, ***P < 0.001. Fig. S3. Verifying the Trlae1 locus in the selected transformants obtained by co‐transformation of different flanking sequences and RNPs. (A) Transformants obtained using 1000 bp flanking sequences and 25 min incubation at 20°C. (B) Transform [file MBT2-14-2343-s001.docx]

**Supporting Information Appendix for:**

**Efficient genome editing in filamentous fungi via an improved CRISPR-Cas9 ribonucleoprotein method facilitated by chemical reagents**

Gen Zou^1,2†^, Meili Xiao ^1,3†^, Shunxing Chai^1,3^, Zhihua Zhu^1,3^, Ying Wang^2^, Zhihua Zhou^1*^

^1^ CAS-Key Laboratory of Synthetic Biology, CAS Center for Excellence in Molecular Plant Sciences, Institute of Plant Physiology and Ecology, Chinese Academy of Science, Fenglin Rd 300, Shanghai 200032, China.

^2^ Shanghai Key Laboratory of Agricultural Genetics and Breeding; Institute of Edible Fungi, Shanghai Academy of Agriculture Science, 1000 Jinqi Rd, Fengxian 201403, Shanghai, China

^3^ University of Chinese Academy of Sciences, Beijing 100049, China.

^†^Gen Zou and Meili Xiao contributed equally to this work

Authors Email:

Gen Zou: [zougen@sibs.ac.cn](mailto:zougen@sibs.ac.cn)

Meili Xiao: [xiaomeili@sippe.ac.cn](mailto:xiaomeili@sippe.ac.cn)

Shunxing Chai: [sxchai@sibs.ac.cn](mailto:sxchai@sibs.ac.cn)

Zhihua Zhu: [zhzhu2016@sibs.ac.cn](mailto:zhzhu2016@sibs.ac.cn)

Ying Wang: wangyingmush@saas.sh.cn

^*^Correspondence to Zhihua Zhou

Email: zhouzhihua@sippe.ac.cn

Tel: +862154924050

Fax: +862154924049


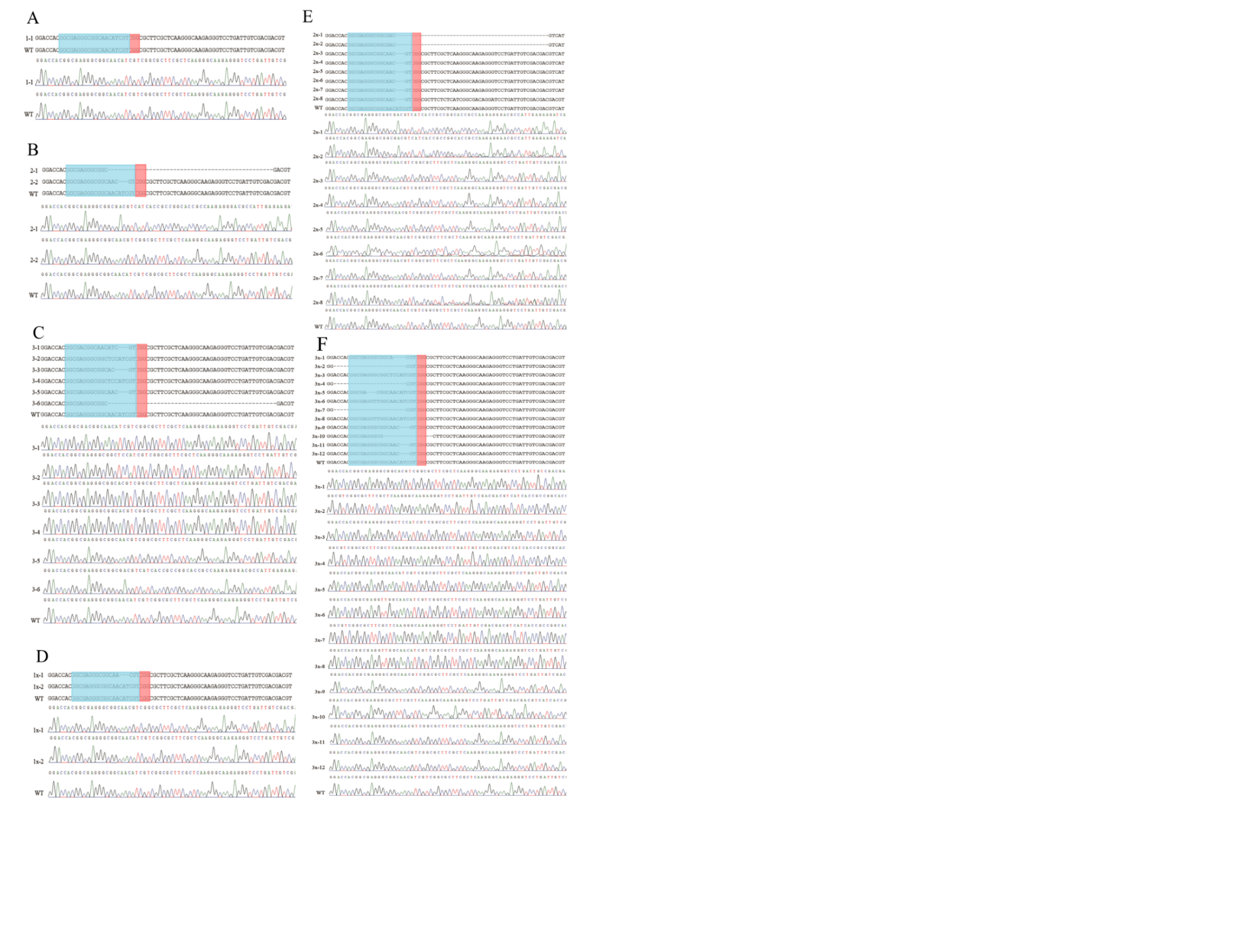


**Figure S1. Verifying *Trura5* mutation after RNP-based gene disruption.** Chromatogram and alignment of *Trura5* sequences of *T. reesei* and its mutants obtained by transformation with 60 nM RNPs **(A)**, 100 nM RNPs **(B)**, 170 nM RNPs **(C) or** 60 nM RNPs and facilitated using 0.006% Triton X-100 **(D)**, 100 nM RNPs loaded and facilitated using 0.006% Triton X-100 **(E)**, 170 nM RNPs loaded and facilitated using 0.006% Triton X-100 **(F)**. WT: wild type strain Rut-C30. 1-1, 2-1, 2-2, and 3-1~3-6: mutants generated by directly transformation of RNPs. 1x-1, 1x-2, 2x-1~2x-8 and 3x-1~3x-12: mutants stimulated by RNP facilitated by 0.006% Triton X-100.


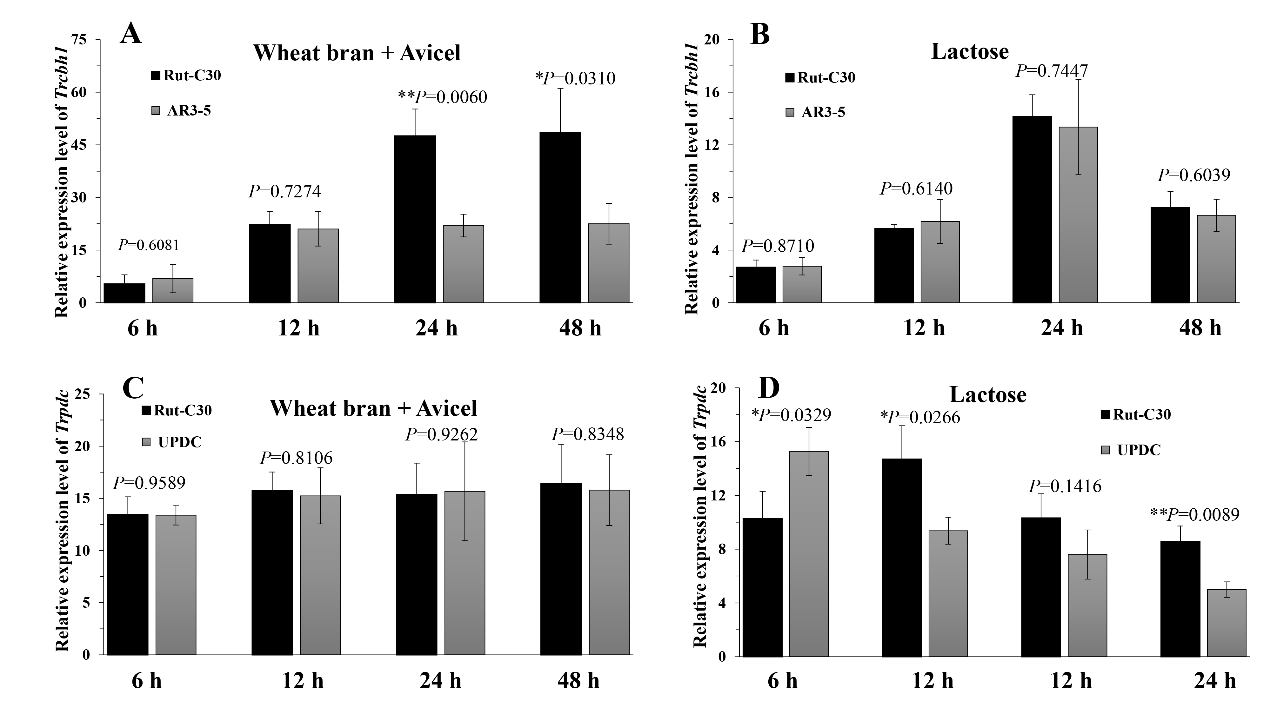


**Figure S2. Relative expression levels of *Trcbh1* and *Trpdc* in Rut-C30 and its deriving strain AR3-5 and UPDC. (A)** Relative expression levels of the endogenous *Trcbh1* in AR3-5 harboring a Cas9 expressed under the control of the inducing promoter Pcbh1 when induced by 2% (w/v) wheat bran plus 3% (w/v) Avicel. **(B)** Relative expression levels of *Trcbh1* in AR3-5 induced by 1% (w/v) lactose. In AR3-5, *Trcbh1* was downregulated 24 h and 48 h after induction of wheat bran and Avicels. **(C)** Relative expression levels of the endogenous *Trpdc* in UPDC harboring a Cas9 expressed under the control of the constitutive promoter Ppdc when induced by 3% (w/v) wheat bran plus 2% (w/v) Avicel. **(D)** Relative expression levels of *Trpdc* in UPDC induced by 1% (w/v) lactose. In UPDC, *Trpdc* was upregulated at 6 h and downregulated at 24 h and 48 h following induction by lactose. *Trsar1* (Trire2|61470) was used as the reference gene. The primers used are listed in (**Table S1**). Error bars show the standard deviation of three replicates. All *P* values were generated from two-tailed *t*-tests using Microsoft Excel: *, *P* < 0.05, **, *P* < 0.01, ***, *P* < 0.001.


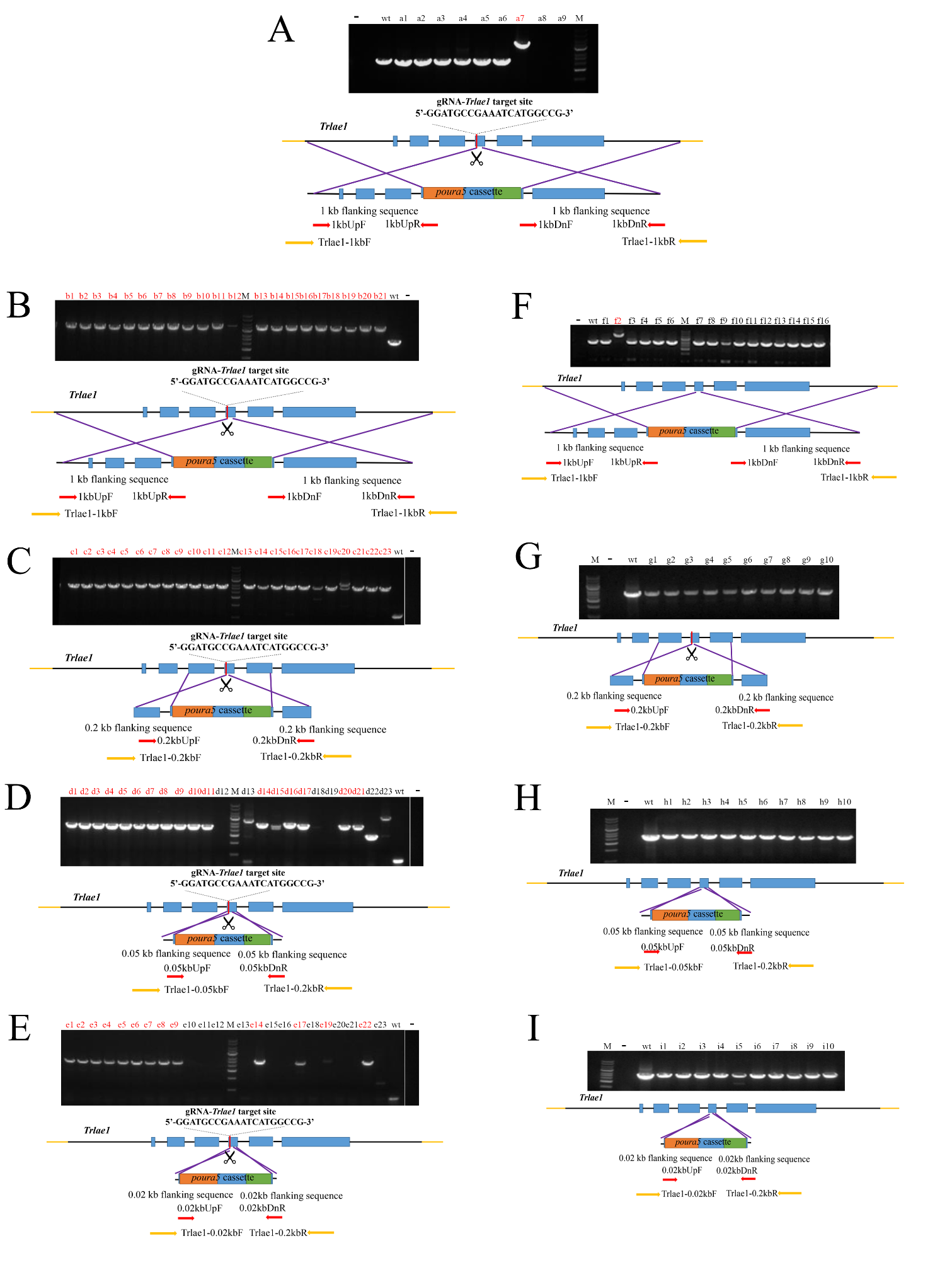


**Figure S3. Verifying the *Trlae1* locus in the selected transformants obtained by co-transformation of different flanking sequences and RNPs. (A)** Transformants obtained using 1000 bp flanking sequences and 25 min incubation at 20°C. **(B)** Transformants obtained using 1000 bp flanking sequences. **(C)** Transformants obtained using 200 bp flanking sequences. **(D)** Transformants obtained using 50 bp flanking sequences. **(E)** Transformants obtained using 20 bp flanking sequences. (**F–I**) Control transformation performed using various flanking sequences without RNPs. All 16 transformants obtained using 1000 bp flanking sequences were verified by PCR (**F**). However, 10 randomly selected transformants obtained from each control transformation using 200 bp (**G**), 50 bp (**H**) or 20 bp (**I**) flanking sequences were verified, as none of these transformants formed white colonies **(Figure S4)**.

The numbers preceded by lowercase letters represent independent transformants from each transformation. M: marker. wt: parent strain 3x-1. -: negative control (negative controls in **C**, **D**, **E** were cut from another gel due to limited numbers of gel wells). Transformants marked with red numbers were repaired by HDR. Red arrows: primers used to amplify the donor DNAs. Yellow arrows: primers used for verification. All primers are listed in **Table S1**.


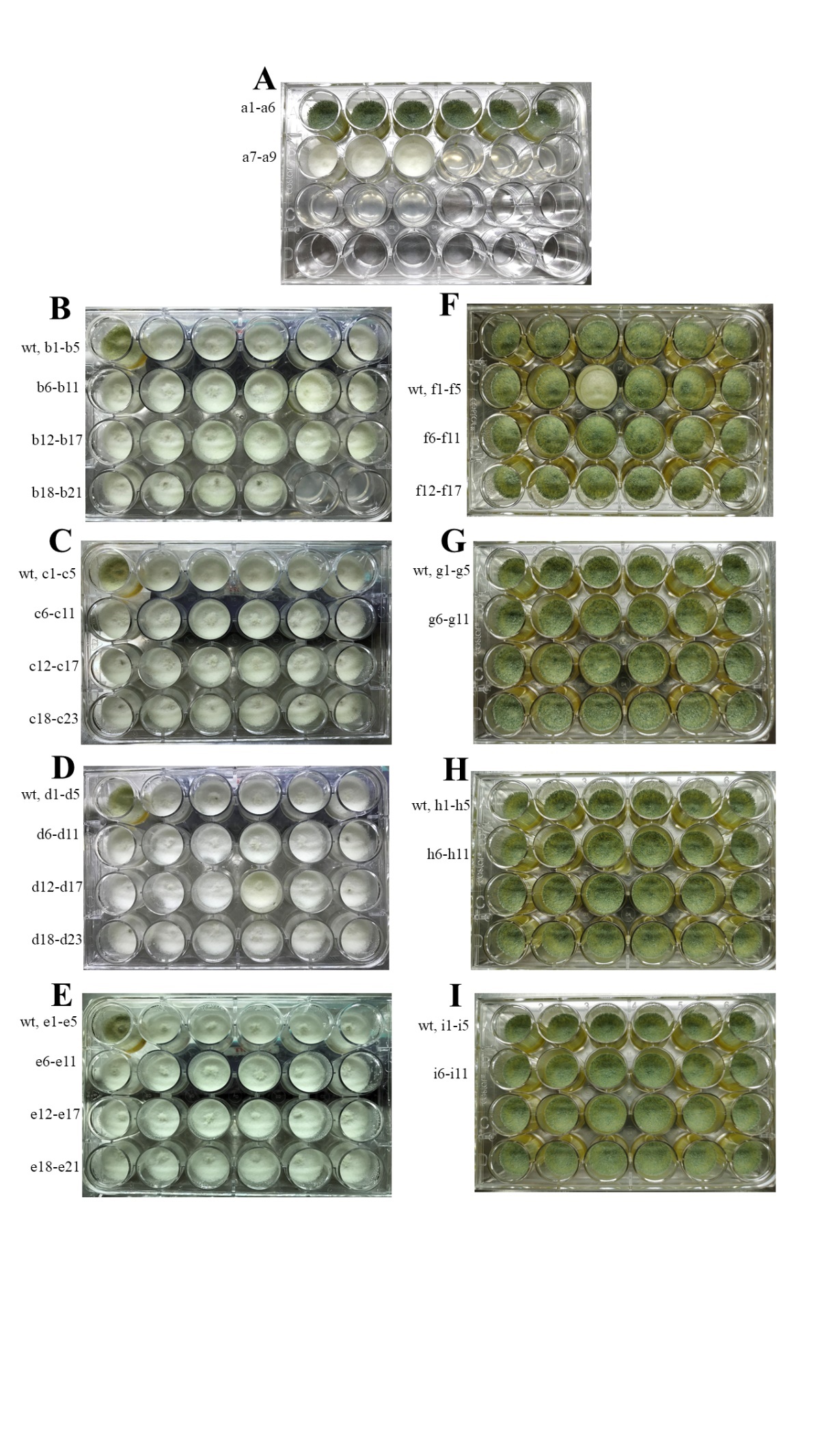


**Figure S4. Morphology of selected transformants with co-transformation of different flanking sequences and RNP directing *Trlae1* locus.** The transformants in each panel correspond one by one to these in **Figure S3**. The numbers that start with lowercase letters represent independent transformants of each transformation. wt: parent strain 3x-1.


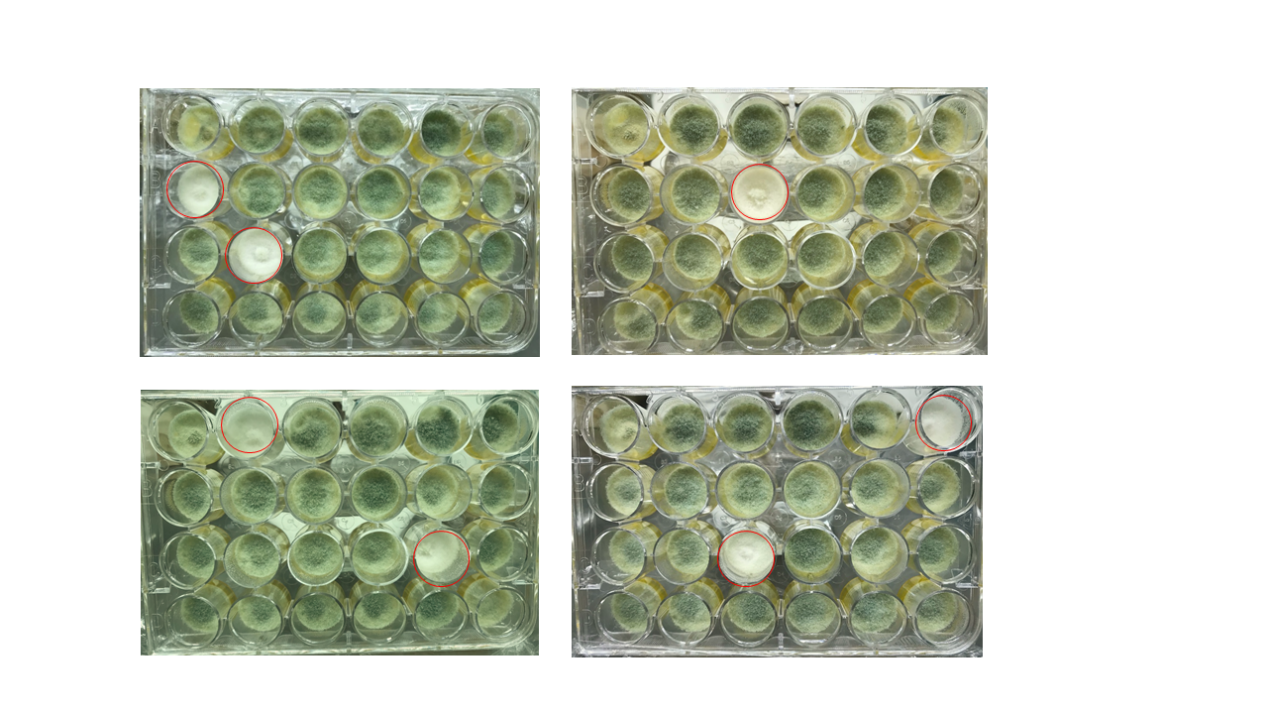


**Figure S5. Morphology of *Trlae1* disruption based on RNP transformation without DNA fragment and selectable marker.** Randomly selected transformants on 24-well plates with PDA medium. Transformants lost *Trlae1* function were marked with red circles.


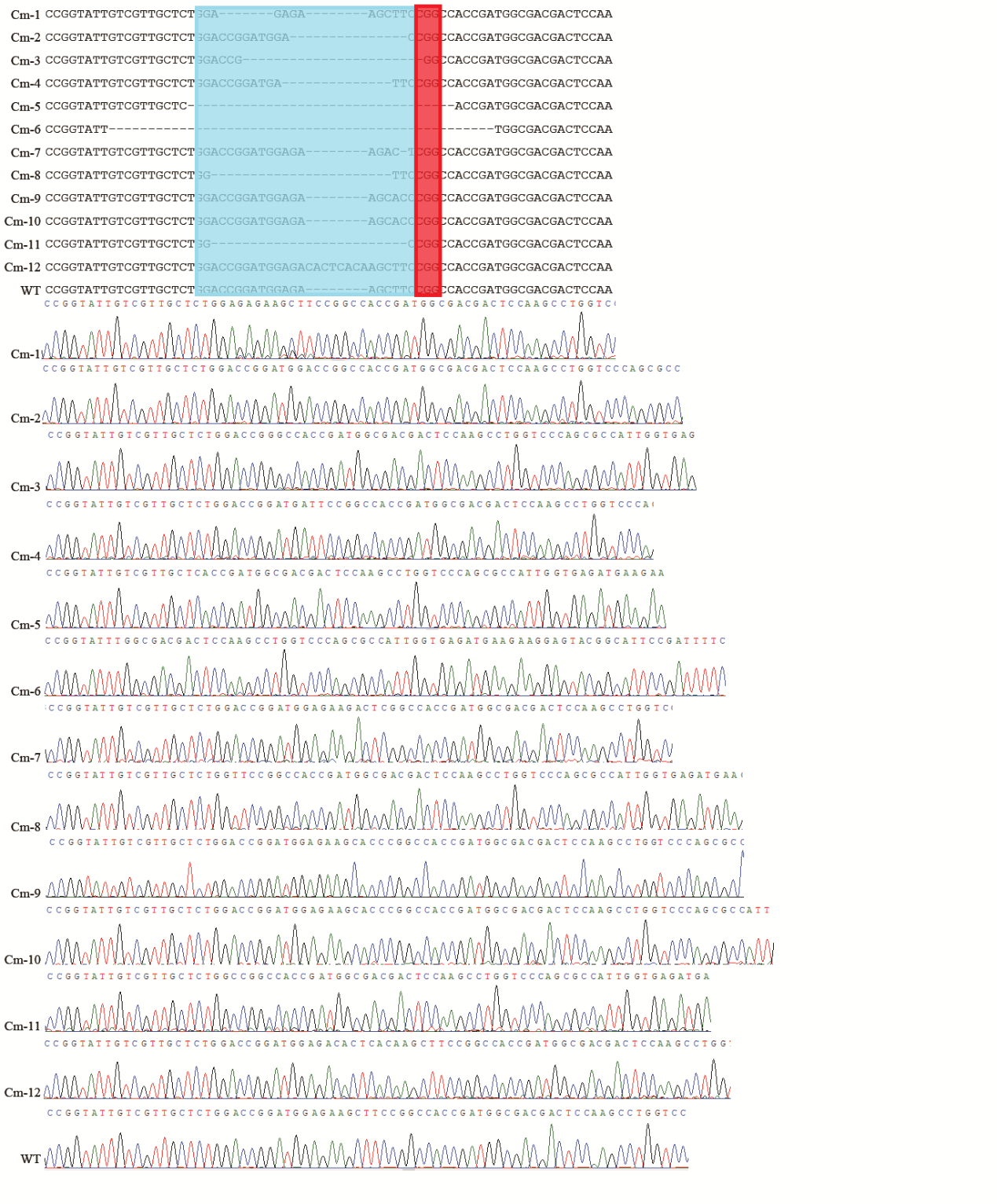


**Figure S6. Verifying *Cmura5* mutation after RNP-based gene disruption when 170 nM RNP loaded and facilitated using 0.006% Trition X-100.** All 12 randomly selected transformants were detected NHEJ in the targeting sequence. WT: wild type *C. militaris* Cm01. Cm-1~Cm-12: randomly selected mutants directed by the optimized transformation procedure facilitated using Triton X-100.


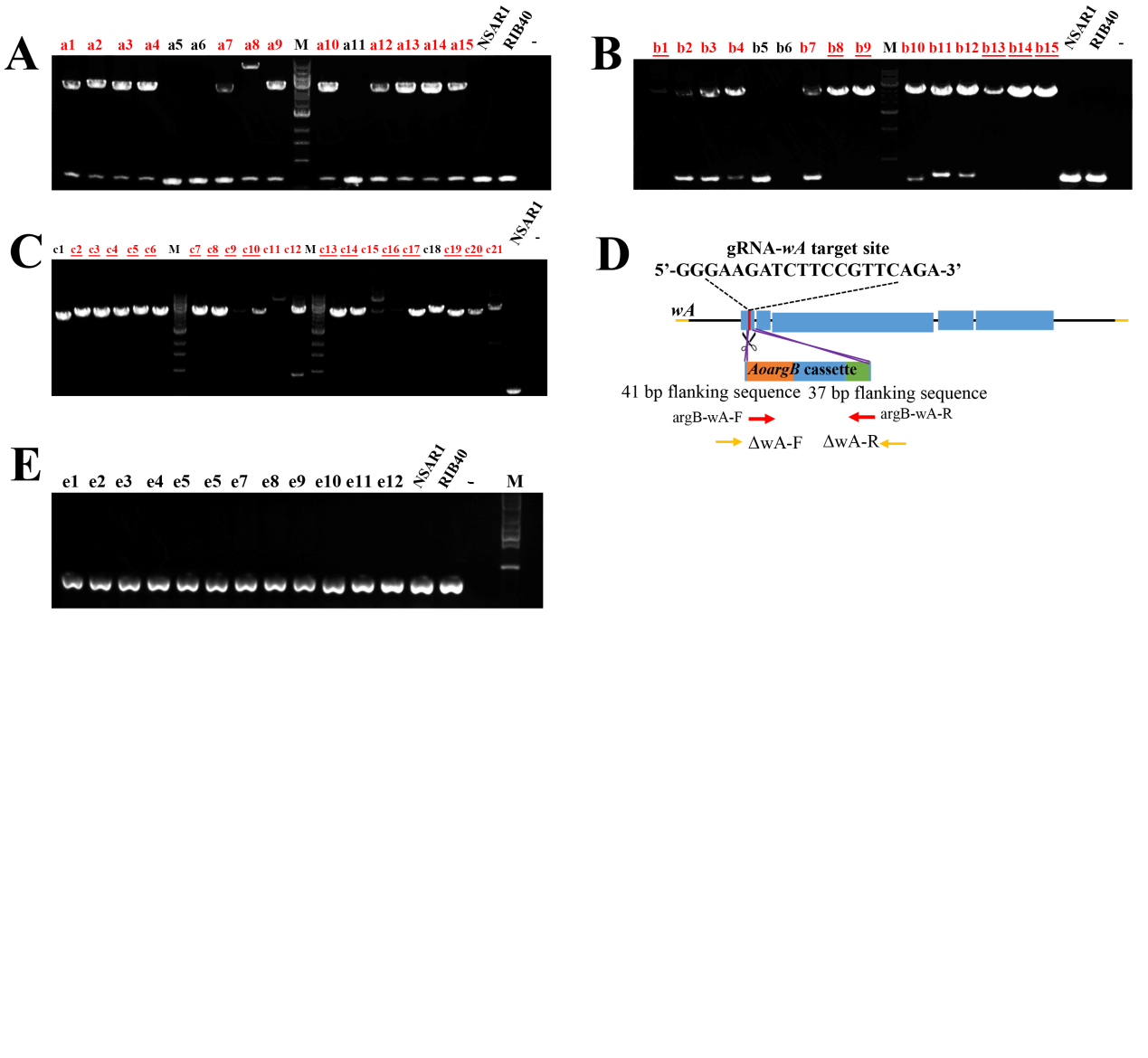


**Figure S7. Verifying the** ***AowA* disruption in the selected transformants obtained by co-transformation of RNPs and *AoargB* selectable marker. (A)** Verification PCR result for transformation performed with Triton X-100. **(B)** Transformation performed with Triton X-100 and 100 mg/L inositol in protoplast preparation. **(C)** Transformation performed with Triton X-100 and 1 mg/L benomyl in protoplast preparation. (**D**) Schematic diagram for the *AowA* disruption. **(E)** Transformants of control transformation performed as **(C)**, but without RNP. The numbers preceded by lowercase letters represent independent transformants from each transformation. M: marker. NSAR1: parent strain in this assay. RIB40, initial parent strain of NSAR1. -: negative control. Transformants marked with red numbers were repaired by HDR. Homozygous transformants were underlined. Red arrows: primers used to amplify donor DNAs. Yellow arrows: primers used for verification. All primers are listed in **Table S1**.


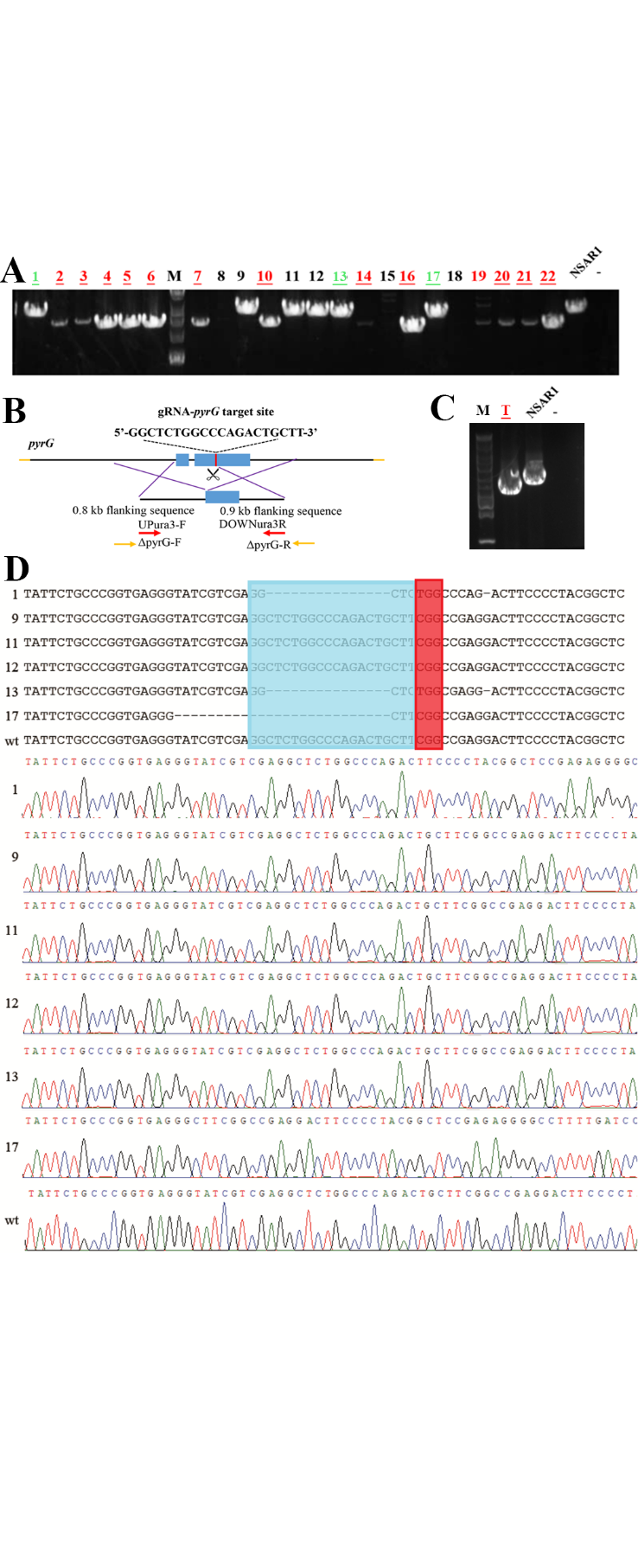


**Figure S8. Verification and sequencing for the *AopyrG* disruption.** Verification PCR **(A)** and schematic diagram **(B)** for the *AopyrG* disruption which was performed using 1 mg/L benomyl and was incubated for 50 min at 20°C. **(C)** Control transformation was performed without RNP. The numbers on the gel pictures represented different transformants from the optimized transformation. M: marker. NSAR1: parent strain. -: negative control. T: the transformant obtained in the control transformation. Transformants marked with red characters were repaired by HDR, while these marked with green characters were repaired by NHEJ. Homozygous transformants were underlined. Red arrows: primers used to amplify donor DNAs. Yellow arrows: primers used for verification. All the primers are listed in **Table S1**. **(D)** Sequencing for the *AopyrG* gene of transformants without detectable HDR. Transformants 1, 13, 17 were detected NHEJ in the targeting sequence. And there was no mutation observed in *AopyrG* gene of transformants 9, 11, 12.


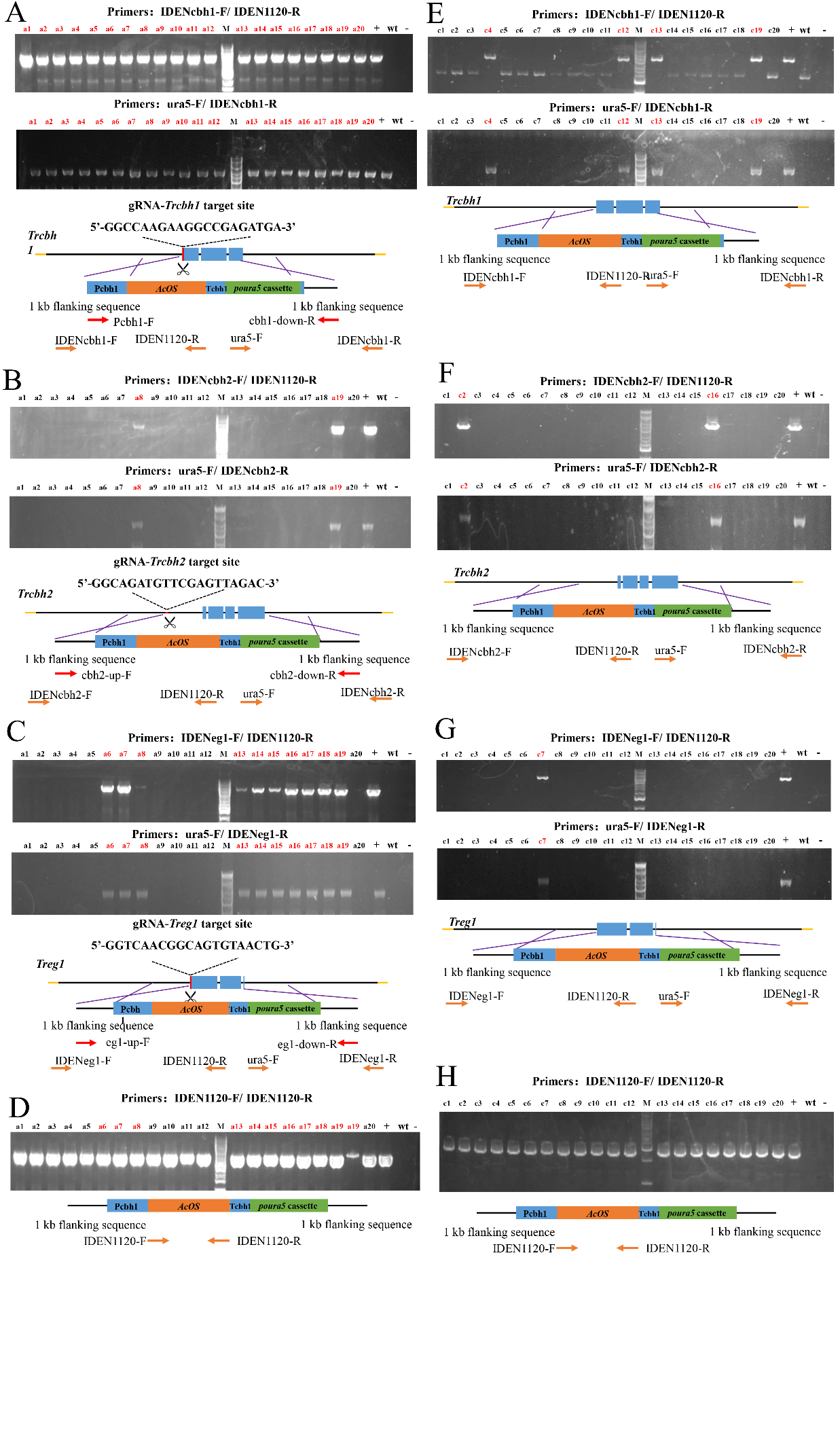


**Figure S9. Verifying *AcOS* insertion targeting *Trcbh1*, *Trcbh2*, *Treg1* in the selected *T. reesei* transformants from co-transformation of RNPs and *poura5* selectable marker. (A) ~ (D)**: Verification PCR for transformation performed using the optimized procedure with Triton X-100. Verifying HDR at the *Trcbh1* **(A)**, *Trcbh2* **(B)**, and *Treg1* **(C)** loci in *T. reesei* transformants. **(D)** Verifying *AcOS* gene in genome of *T. reesei* transformants. Two of 20 transformants (10.0%, transformants a8 and a19) had correct donor DNA insertion at all of the genomic targets. Eight of them (40.0%, transformants a6, a7, a13, a14, a15, a16, a17 and a18) were conducted two sites (*Trcbh1*, *Treg1*) genome edited. And all the rest transformants (50.0%, transformants a1, a2, a3, a4, a5, a9, a10, a11, a12 and a20) replaced the *Trcbh1* gene. **(E) ~ (H)**: Verification PCR for control transformation performed without RNPs. Three of 20 randomly selected transformants had HDR at *Trcbh1* locus **(E)**. Two transformants had replaced *Trcbh2* gene **(F)**. One transformant was conducted *Treg1* edited correctly **(G)**. *AcOS* insertion could be detected in all transformants obtained by the control transformation **(H)**. However, the HDR efficiency (5%~15%) was very low without RNP. None had multiplexed homologous recombination at the targeting genomic targets.

The numbers preceded by lowercase letters represent independent transformants from each transformation. M: marker. wt: 3x-1 as the parent strain in this assay. +: positive control. -: negative control. Transformants marked with red numbers were repaired by HDR pathway. Red arrows: primers used to amplify donor DNAs. Yellow arrows: primers used for verification. All the primers are listed in **Table S1**.


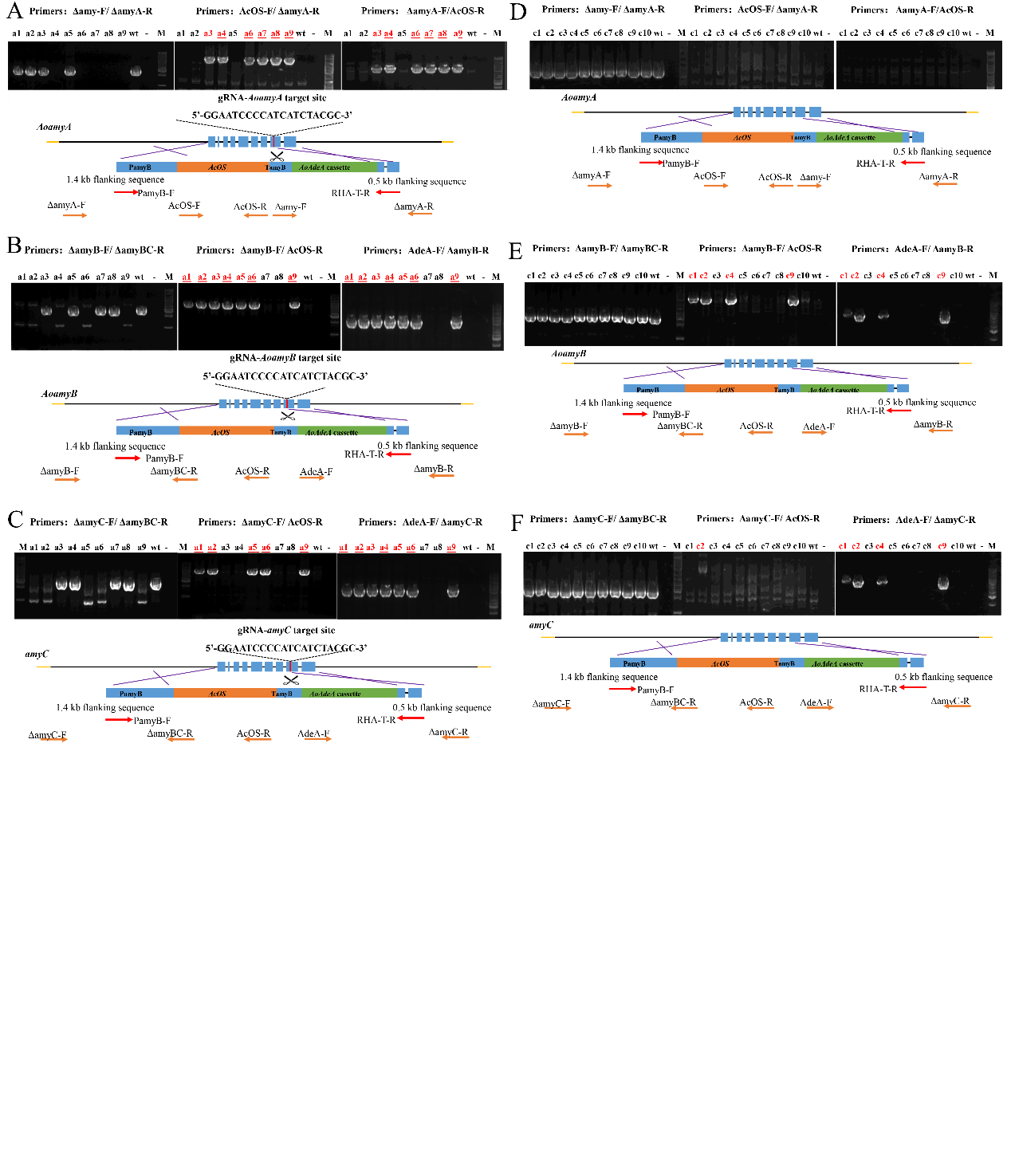


**Figure S10. Verifying *AcOS* insertion targeting *AoamyA*, *AoamyB*, *AoamyC* in the selected *A. oryzae* transformants from co-transformation of RNPs and *AoadeA* selectable marker. (A) ~ (C)** Verification PCR for transformation performed using the optimized procedure with Triton X-100 and benomyl. Verifying HDR at *AoamyA* **(A)**, *AoamyC* **(B)**, and *AoamyC* **(C)** loci in *A. oryzae* transformants. Two of 9 transformants (22.2%, transformants a6 and a10) had correct donor DNA insertion at all of the genomic targets. Five of them (55.6%) were conducted two genome-edited sites (transformants a1, a2 and a5 at *AoamyB* and *AoamyC*, transformants a3 and a4 at *AoamyA* and *AoamyB*). And all the rest transformants (22.2%, transformants a7 and a8) replaced the *AoamyA* gene by *AcOS*. Except transformants a3 and a5, the rest transformants were homozygous transformants. Since the same 3’ flanking sequence (> 9 kb) shared with *AoamyB* and *AoamyC*, PCR was performed using primers AdeA-F/ΔamyB-R **(B)** and AdeA-F/ΔamyC-R **(C)**. **(D) ~ (F)** Verification PCR for the control transformation performed without RNP. None of 10 transformants had HDR at *AoamyA* **(D)**. Four transformants (c1, c2, c4 and c9) replaced *AoamyB* gene **(E)**. One transformant was replaced *AoamyC* gene **(F)**. Due to the same 3’ flanking sequence, PCR results were shown in the same gel picture verified using primers AdeA-F/ΔamyB-R **(E)** and AdeA-F/ΔamyC-R **(F)**. One of them (transformant c2) exhibited the Δ*AoamyB* and Δ*AoamyC* karyotypes. However, none of them was homozygous transformant in the control transformation.

The numbers preceded by lowercase letters represent independent transformants from each transformation. M: marker. wt: NSAR1 as the parent strain in this study. -: negative control. Transformants marked with red numbers were repaired by HDR. Homozygous transformants are underlined. Red arrows: primers used to amplify donor DNAs. Yellow arrows: primers used for verification. All the primers are listed in Table S1.

**Supplementary Information Tables**

**Table S1 Oligonucleotides and gene sequences used in this study**

| **Names of oligonucleotides and genes** | **Sequences (5'-3')** |
| --- | --- |
| gRNA_M_*Trura5* | *taatacgactcactatagg*cgagggcggcaacatcgtgttttagagctagaaatagcaagttaaaataaggctagtccgttatcaacttgaaaaagtggcaccgagtcggtgctttt (Target sequence of *Trura5* is underlined. T7 promoter is in *italics*. Sequence of gRNA_*Trura5* is shown in green) |
| gRNA_M_*Trlae1* | *taatacgactcactatagg*atgccgaaatcatggccggttttagagctagaaatagcaagttaaaataaggctagtccgttatcaacttgaaaaagtggcaccgagtcggtgctttt (Target sequence of *Trlae1* is underlined. T7 promoter is in *italics*. Sequence of gRNA_*Trura5* is shown in green) |
| gRNA_M_*Cmura5* | *taatacgactcactatagg*accggatggagaagcttcttttagagctagaaatagcaagttaaaataaggctagtccgttatcaacttgaaaaagtggcaccgagtcggtgctttt (Target sequence of *Cmura5* is underlined. T7 promoter is in *italics*. Sequence of gRNA_*Cmura5* is shown in green) |
| gRNA_M_*Trcbh1* | *taatacgactcactatagg*ccaagaaggccgagatgagttttagagctagaaatagcaagttaaaataaggctagtccgttatcaacttgaaaaagtggcaccgagtcggtgctttt (Target sequence of *Trcbh1* is underlined. T7 promoter is in *italics*. Sequence of gRNA_*Trcbh1* is shown in green) |
| gRNA_M_*Trcbh2* | *taatacgactcactatagg*cagatgttcgagttagacgttttagagctagaaatagcaagttaaaataaggctagtccgttatcaacttgaaaaagtggcaccgagtcggtgctttt (Target sequence of *Trcbh2* is underlined. T7 promoter is in *italics*. Sequence of gRNA_*Trcbh2* is shown in green) |
| gRNA_M_*Treg1* | *taatacgactcactatagg*tcaacggcagtgtaactggttttagagctagaaatagcaagttaaaataaggctagtccgttatcaacttgaaaaagtggcaccgagtcggtgctttt (Target sequence of *Treg1* is underlined. T7 promoter is in *italics*. Sequence of gRNA_*Treg1* is shown in green) |
| gRNA_M_*AowA* | *taatacgactcactataggg*aagatcttccgttcagagttttagagctagaaatagcaagttaaaataaggctagtccgttatcaacttgaaaaagtggcaccgagtcggtgctttt (Target sequence of *AowA* is underlined. T7 promoter is in *italics*. Sequence of gRNA_*AowA* is shown in green) |
| gRNA_M_*AopyrG* | *taatacgactcactatagg*ctctggcccagactgcttgttttagagctagaaatagcaagttaaaataaggctagtccgttatcaacttgaaaaagtggcaccgagtcggtgctttt (Target sequence of *AopyrG* is underlined. T7 promoter is in *italics*. Sequence of gRNA_*AopyrG* is shown in green) |
| gRNA_M_*Aoamy* | *taatacgactcactatagg*aatccccatcatctacgcgttttagagctagaaatagcaagttaaaataaggctagtccgttatcaacttgaaaaagtggcaccgagtcggtgctttt (Target sequence of *AoamyA*, *AoamyB*, *AoamyC* is underlined. T7 promoter is in *italics*. Sequence of gRNA_*Aoamy* is shown in green) |
| AcOS_optimized_for_*T._reesei* | ATGGCCTGCAAGTACAGCACCCTCATCGACAGCAGCCTCTACGACCGCGAGGGCCTCTGCCCTGGCATCGACCTCCGCCGCCACGTCGCTGGCGAGCTTGAGGAGGTCGGCGCCTTTCGCGCCCAGGAGGACTGGCGCCGACTCGTCGGCCCTCTGCCTAAGCCTTACGCCGGCCTGCTCGGCCCCGACTTCAGCTTCATCACCGGCGCTGTCCCCGAGTGCCATCCTGACCGCATGGAGATCGTCGCCTACGCTCTCGAGTTCGGCTTCATGCACGACGACGTCATCGACACCGACGTCAACCACGCCAGCCTCGACGAGGTCGGCCACACGCTCGACCAGAGCCGCACCGGCAAGATCGAGGACAAGGGCAGCGACGGCAAGCGCCAGATGGTCACCCAGATCATCCGAGAGATGATGGCTATCGACCCCGAGCGCGCCATGACCGTCGCCAAGAGCTGGGCCAGCGGCGTCCGCCACAGCAGCCGCCGCAAGGAGGACACCAACTTCAAGGCCCTCGAGCAGTACATCCCCTACCGCGCTCTGGACGTCGGCTACATGCTCTGGCACGGCCTCGTCACCTTCGGCTGCGCCATCACGATCCCCAACGAGGAGGAGGAGGAGGCCAAGCGCCTCATCATCCCCGCTCTCGTCCAGGCCAGCCTCCTCAACGACCTGTTCAGCTTCGAGAAGGAGAAGAACGACGCCAACGTCCAGAACGCCGTCCTCATCGTCATGAACGAGCACGGCTGCAGCGAGGAGGAGGCTCGCGACATCCTCAAGAAGCGCATCCGCCTCGAGTGCGCCAACTACCTCCGCAACGTCAAGGAGACTAACGCTCGCGCCGACGTGAGCGACGAGCTGAAGCGCTACATCAACGTCATGCAGTACACCCTCAGCGGCAACGCCGCCTGGTCCACGAACTGCCCTCGCTACAACGGCCCCACCAAGTTCAACGAGCTTCAGCTGCTCCGCTCGGAGCACGGCCTGGCTAAGTACCCCAGCCGCTGGTCGCAGGAGAACCGCACCAGCGGCCTTGTCGAGGGCGACTGCCACGAGAGCAAGCCTAACGAGCTTAAGCGAAAGCGCAACGGCGTCAGCGTCGACGACGAGATGCGCACCAACGGCACGAACGGCGCCAAGAAGCCCGCTCACGTTTCGCAGCCCAGCACCGACAGCATCGTGCTCGAGGACATGGTCCAGCTCGCCCGAACCTGCGACCTGCCTGACCTCAGCGACACCGTCATCCTCCAGCCTTACCGCTACCTCACCTCGCTGCCCAGCAAGGGCTTCCGCGACCAGGCCATCGACTCCATCAACAAGTGGCTCAAGGTCCCTCCTAAGAGCGTCAAGATGATCAAGGACGTCGTGAAGATGCTCCACAGCGCCAGCCTGATGCTCGACGACCTCGAGGATAACTCGCCCCTCCGCCGAGGCAAGCCCTCCACGCACAGCATCTACGGCATGGCCCAGACCGTCAACAGCGCCACCTACCAGTACATCACCGCCACCGACATCACGGCCCAGCTGCAGAACAGCGAGACTTTCCACATCTTCGTCGAGGAGCTGCAGCAGCTGCACGTCGGCCAGAGCTACGACCTCTACTGGACCCACAACACGCTCTGCCCCACGATCGCCGAGTACCTCAAGATGGTCGACATGAAGACTGGCGGCCTCTTCCGCATGCTCACCCGCATGATGATCGCTGAGAGCCCTGTCGTCGACAAGGTCCCCAACAGCGACATGAACCTCTTCTCGTGCCTGATCGGCCGCTTCTTCCAGATCCGCGACGACTACCAGAACCTGGCCAGCGCCGACTACGCCAAGGCCAAGGGCTTTGCCGAGGACCTGGATGAGGGCAAGTACTCGTTCACCCTGATCCACTGCATCCAGACGCTCGAGTCGAAGCCTGAGCTGGCCGGCGAGATGATGCAGCTCCGCGCCTTCCTGATGAAGCGCCGACACGAGGGCAAGCTCAGCCAGGAGGCTAAGCAGGAGGTCCTGGTCACCATGAAGAAGACCGAGAGCCTCCAGTACACGCTCTCCGTCCTCCGCGAGCTGCACTCGGAGCTTGAGAAGGAGGTCGAGAACCTCGAGGCCAAGTTCGGCGAGGAGAACTTCACCCTCCGCGTCATGCTCGAGCTGCTGAAGGTCTAA |
| AcOS_optimized_for_*A._oryza****e*** | ATGGCCTGCAAGTACAGCACCCTCATCGATTCCAGCCTCTACGATCGCGAGGGTCTGTGCCCTGGCATCGATCTCCGCCGCCATGTCGCCGGTGAGCTGGAAGAGGTCGGCGCCTTCCGCGCTCAAGAGGATTGGCGTCGCCTCGTCGGCCCTCTGCCTAAGCCTTACGCCGGCCTGCTCGGCCCTGATTTCTCCTTCATCACCGGCGCTGTCCCTGAGTGCCATCCTGATCGCATGGAAATCGTCGCCTACGCTCTCGAGTTCGGCTTCATGCACGATGATGTCATCGATACCGATGTCAACCATGCCAGCCTCGATGAGGTCGGTCATACCCTCGATCAGTCCCGCACCGGCAAGATCGAGGATAAGGGCTCCGATGGCAAGCGCCAGATGGTCACCCAGATCATCCGCGAAATGATGGCTATCGATCCTGAGCGCGCCATGACCGTCGCCAAGTCCTGGGCCTCCGGCGTCCGCCATTCCAGCCGCCGCAAAGAGGATACCAACTTCAAGGCCCTCGAGCAGTACATCCCTTACCGCGCTCTGGATGTCGGCTACATGCTCTGGCATGGCCTCGTCACCTTCGGCTGCGCCATCACTATCCCTAACGAGGAAGAGGAAGAAGCCAAGCGCCTCATCATCCCTGCTCTCGTCCAGGCCAGCCTCCTCAACGATCTCTTCAGCTTCGAGAAAGAGAAGAACGACGCCAACGTCCAGAACGCCGTCCTTATCGTCATGAACGAGCATGGCTGCTCCGAAGAAGAGGCCCGCGATATCCTCAAGAAGCGCATCCGCCTCGAGTGCGCCAACTACCTCCGCAACGTCAAAGAGACTAACGCTCGCGCCGATGTCTCTGATGAGCTGAAGCGCTACATCAACGTCATGCAGTACACCCTCTCCGGCAACGCCGCCTGGTCCACCAACTGTCCTCGCTACAACGGCCCTACCAAGTTCAACGAGCTTCAGCTGCTCCGCTCCGAGCACGGCCTCGCTAAGTACCCTAGCCGCTGGTCCCAAGAGAACCGCACCTCCGGCCTCGTCGAGGGCGATTGCCATGAGTCCAAGCCTAACGAGCTTAAGCGCAAGCGCAACGGCGTCAGCGTCGATGATGAGATGCGCACCAACGGCACCAATGGCGCCAAGAAGCCTGCTCACGTTTCCCAGCCTTCCACCGATTCCATCGTGCTCGAGGATATGGTCCAGCTCGCCCGCACCTGTGATCTCCCTGATCTCTCCGATACCGTCATCCTCCAGCCTTACCGCTACCTCACCAGCCTGCCTTCCAAGGGCTTCCGCGATCAGGCCATCGATTCTATCAACAAGTGGCTCAAGGTCCCTCCTAAGTCCGTCAAGATGATCAAGGATGTCGTGAAGATGCTCCATTCCGCCAGCCTCATGCTGGATGATCTCGAGGACAACTCCCCTCTGCGCCGTGGCAAGCCCTCCACTCATTCCATCTACGGCATGGCCCAGACCGTCAACTCCGCCACCTACCAGTACATCACCGCCACCGATATCACCGCTCAGCTCCAGAACTCCGAAACCTTCCATATCTTCGTCGAGGAACTCCAGCAGCTCCATGTCGGCCAGTCCTACGATCTCTACTGGACCCATAACACCCTCTGTCCTACTATCGCCGAGTACCTCAAGATGGTCGATATGAAGACCGGTGGCCTCTTCCGCATGCTCACCCGGATGATGATCGCTGAGTCCCCTGTCGTCGATAAGGTCCCTAACTCCGATATGAACCTCTTCTCCTGCCTGATCGGCCGCTTCTTCCAGATCCGCGACGATTACCAGAACCTCGCCTCCGCCGATTACGCCAAGGCCAAGGGTTTCGCCGAGGATCTGGATGAGGGCAAGTACTCTTTCACCCTGATCCATTGCATCCAGACTCTCGAGTCTAAGCCTGAGCTGGCCGGCGAGATGATGCAGCTCCGCGCCTTCCTGATGAAGCGCCGCCACGAGGGCAAGCTCTCCCAAGAGGCCAAGCAAGAGGTCCTGGTCACCATGAAGAAAACCGAGAGCCTCCAGTATACCCTGTCCGTCCTCCGCGAGCTGCATTCCGAGTTGGAGAAAGAGGTCGAGAACCTCGAGGCCAAGTTCGGCGAGGAAAACTTCACCCTCCGCGTCATGCTCGAGCTGCTGAAGGTCTAA |
| rtTrsar1F | TGGATCGTCAACTGGTTCTACGA (Primer for reference gene *Trsar1* in quantitative real-time PCR) |
| rtTrsar1R | GCATGTGTAGCAACGTGGTCTTT (Primer for reference gene *Trsar1* in quantitative real-time PCR) |
| rtTrcbh1F | GTCTGTGGGATGATTACTACG (Primer for detecting expression of *Trcbh1* in quantitative real-time PCR) |
| rtTrcbh1R | CCGAACTTGATGTTGGAGAAG (Primer for detecting expression of *Trcbh1* in quantitative real-time PCR) |
| rtTrpdcF | TTCAACACTGCGGGCTTCT (Primer for detecting expression of *Trpdc* in quantitative real-time PCR) |
| rtTrpdcR | CAGAACACCCCTCATCGCTAC(Primer for detecting expression of *Trpdc* in quantitative real-time PCR) |
| 1kbUpF | GAAGCCAATCAGCAAGGTTG (Primer for amplifying the 5′ flanking region (1 kb) for HDR of *Trlae1* and the dDNA targeting *Trlae1* with 1 kb flanking sequence using dDNAlae1-1k as template) |
| 1kbUpR | CTGGCTTAACTATGCGGC TTGGCATACCTGAAAAATGTG (Primer for amplifying the 5′ flanking region (1 kb) for HDR of *Trlae1*) |
| 1kbDnF | GAAAATTCCGTCACCAGCCCTG CGGCAGCATAAGAGATGACAAG (Primer for amplifying the 3′ flanking region (1 kb) for HDR of *Trlae1* and the dDNA targeting *Trlae1* with 1 kb flanking sequence using dDNAlae1-1k as template) |
| 1kbDnR | GTGCTTCATTCGAATGAGAC (Primer for amplifying the 3′ flanking region (1 kb) for HDR of *Trlae1*) |
| UF | GCCGCATAGTTAAGCCAG (Primer for amplifying *ura5* cassette for screening of *Trlae1* deletion strain) |
| UR | CAGGGCTGGTGACGGAATTTTC (Primer for amplifying *ura5* cassette for screening of *Trlae1* deletion strain) |
| 0.2kbUpF | ATGTCTTTCACAAGTATTTC (Primer for amplifying dDNA targeting *Trlae1* with 0.2 kb flanking sequence using dDNAlae1-1k as template) |
| 0.2kbDnR | TGAAGAGCCGAATGTCTG (Primer for amplifying dDNA targeting *Trlae1* with 0.2 kb flanking sequence using dDNAlae1-1k as template) |
| 0.05kbUpF | TAGTTTCACACTACGCTGTG (Primer for amplifying dDNA targeting *Trlae1* with 0.05 kb flanking sequence using dDNAlae1-1k as template) |
| 0.05kbDnR | CAAAGGCCTTGCGGTAGACA (Primer for amplifying dDNA targeting *Trlae1* with 0.05 kb flanking sequence using dDNAlae1-1k as template) |
| 0.02kbUpF | ACATTTTTCAGGTATGCCAA (Primer for amplifying dDNA targeting *Trlae1* with 0.02 kb flanking sequence using dDNAlae1-1k as template) |
| 0.02kbDnR | TGTCATCTCTTATGCTGCCG (Primer for amplifying dDNA targeting *Trlae1* with 0.02 kb flanking sequence using dDNAlae1-1k as template) |
| Trlae1-1kbF | ATGAATGCTGTGCTCAGTC (Primer for verifying transformants of *Trlae1* disruption with 1 kb flanking sequence) |
| Trlae1-1kbR | CGTAAAGAACGAAGACGAG (Primer for verifying transformants of *Trlae1* disruption with 1 kb flanking sequence) |
| Trlae1-0.2kbF | ACAACTAACAACATCACCAG (Primer for verifying transformants of *Trlae1* disruption with 0.02 kb, 0.05 kb and 0.2 kb flanking sequence) |
| Trlae1-0.2kbR | TGGTAGCGATGCATGGCAC (Primer for verifying transformants of *Trlae1* disruption with 0.02 kb, 0.05 kb and 0.2 kb flanking sequence) |
| Trura5F | ATGGCTACCACCTCCCAG (Primer for verifying transformants of *Trura5* disruption) |
| Trura5R | GTATGGGTTTTCTCTCTC (Primer for verifying transformants of *Trura5* disruption) |
| Pcbh1-F | CTCGGTACCCGGGGATCCTCTAGAGACTGACCGGACGTGTTTTG (Primer for amplifying the 5′ flanking region (including promoter sequence of *Trcbh1*) for HDR of *Trcbh1*) |
| Pcbh1-R | GCTGTACTTGCAGGCCATGATGCGCAGTCCGCGGTTG (Primer for amplifying the 5′ flanking region (including promoter sequence of *Trcbh1*) for HDR of *Trcbh1*) |
| TrAcOS-F (IDEN1120-F) | ATGGCCTGCAAGTACAGCAC (Primer for amplifying AcOS gene (including His tag) optimized for *T. reesei*) |
| TrAcOS-R | ATGATGATGATGATGATGGACCTTCAGCAGCTCGAG (Primer for amplifying AcOS gene (including His tag) optimized for *T. reesei*) |
| Tcbh1-F | CATCATCATCATCATTAAAGCTCCGTGGCGAAAGCCTG (Primer for amplifying terminator sequence of *Trcbh1*) |
| Tcbh1-R | ACACATTCCACAGAGATCTACTAATTTCCACTGTTGC (Primer for amplifying terminator sequence of *Trcbh1*) |
| ura5-F | AGATCTCTGTGGAATGTGTG (Primer for amplifying *ura5* cassette for screening of AcOS transformants and verification of *Trcbh1*, *Trcbh2*, *and Treg1* deletion) |
| ura5-R | TGCCTGCAGGTCGACGATTCTAGACAGGGCTGGTGACGGAATTTTC (Primer for amplifying *ura5* cassette for screening of AcOS transformants) |
| cbh1-down-F | CTCGGTACCCGGGGATCCTCTAGACCAGTGCGGCGGTATTGG (Primer for amplifying the 3′ flanking region for HDR of *Trcbh1*) |
| cbh1-down-R | TGCCTGCAGGTCGACGATTCTCATTGCGCACTATAG (Primer for amplifying the 3′ flanking region for HDR of *Trcbh1*) |
| cbh2-up-F | CTCGGTACCCGGGGATCCATTACGACTCAAATCAATAC (Primer for amplifying the 5′ flanking region (including promoter sequence of *Trcbh2*) for HDR of *Trcbh2*) |
| cbh2-up-R | CCGACCTGCGACAGACAATCTAGACATGATGAGGGCATCCGTTG (Primer for amplifying the 5′ flanking region (including promoter sequence of *Trcbh2*) for HDR of *Trcbh2*) |
| cbh2-down-F | TTGTCTGTCGCAGGTCGGTAG (Primer for amplifying the 3′ flanking region for HDR of *Trcbh2*) |
| cbh2-down-R | TGCCTGCAGGTCGACGATGAAATTTTTTCTTACCAC (Primer for amplifying the 3′ flanking region for HDR of *Trcbh2*) |
| eg1-up-F | CTCGGTACCCGGGGATCCAGTGTATCAACGAAGTGG (Primer for amplifying the 5′ flanking region (including promoter sequence of *Treg1*) for HDR of *Treg1*) |
| eg1-up-R | AACAATCAGCTGGCATAATCTAGACAAGAAGGACTAAGATAG (Primer for amplifying the 5′ flanking region (including promoter sequence of *Treg1*) for HDR of *Treg1*) |
| eg1-down-F | TTATGCCAGCTGATTGTTTG (Primer for amplifying the 3′ flanking region for HDR of *Treg1*) |
| eg1-down-R | TGCCTGCAGGTCGACGATTTCATCCTCATTGGACTC (Primer for amplifying the 3′ flanking region for HDR of *Treg1*) |
| IDENcbh1-F | ATGGTCATCAAACAAAGAAC(Primer for verification of *Trcbh1* deletion) |
| IDENcbh1-R | GAAGATCAATGCTGTGCAAG (Primer for verification of *Trcbh1* deletion) |
| IDENcbh2-F | GCATGGCTTGCCCATCAC (Primer for verification of *Trcbh2* deletion) |
| IDENcbh2-R | TCGTGGTGAACCAATGGTTC (Primer for verification of *Trcbh2* deletion) |
| IDENeg1-F | ACTTCCTGACCCAGTTTCAG (Primer for verification of *Treg1* deletion) |
| IDENeg1-R | GGTGGCTATGGAGACATTG (Primer for verification of *Treg1* deletion) |
| IDEN1120-R | CGTCCTTGATCATCTTGAC (Primer for verification of *Trcbh1*, *Trcbh2*, *and Treg1* deletion) |
| argB-wA-F | GATGCTTTCACGCTTTGCGCCAAGAGATCGCGAGGCTTTCACCCTGTATCCTGGCCTG (Primer for amplifying dDNA for *AowA* deletion) |
| argB-wA-R | GTCAACGCACTCTCCAGAGCCGGATTAGGGTCTGACTTCGAGATGTTAATGGAGCTAG (Primer for amplifying dDNA for *AowA* deletion) |
| ΔwA-F | AGACCAGACAAGTGATTTCG (Primer for verification of *AowA* deletion strain) |
| ΔwA-R | CGTTTCCAAGGTCTCCGTAG (Primer for verification of *AowA* deletion strain) |
| UP-F | CCCGGGGATCCTCTAGAGATATTTTATTGGCCATCAAAC (Primer for amplifying the 5′ flanking region of *AopyrG*) |
| UP-R | GCCGTAGGGGAAGTCCTCGGGGAAGACATGTTGGCGATG (Primer for amplifying the 5′ flanking region of *AopyrG*) |
| DOWN-F | CCGAGGACTTCCCCTACGGCTC (Primer for amplifying the 3′ flanking region of *AopyrG*) |
| DOWN-R | CATGCCTGCAGGTCGACGATCTGCAGATACAACTGCACC (Primer for amplifying the 3′ flanking region of *AopyrG*) |
| Δ*pyrG*-F | ACTCGAAGTACTAACCTAC (Primer for verification of *AopyrG* deletion strain) |
| Δ*pyrG*-R | TGACGTGTCGAGACGAATC (Primer for verification of *AopyrG* deletion strain) |
| PamyB-F | TCGGTACCTCTAGAATTGTGCAAATGCCGTAAGC (Primer for amplifying the 5′ flanking region (including promoter sequence of *AoamyB*) for HDR of *AoamyA*, *AoamyB*, *AoamyC*) |
| PamyB-R | GATGATGATGGCTAGCAAATGCCTTCTGTGGGGTTTATTG (Primer for amplifying the 5′ flanking region (including promoter sequence of *AoamyB*) for HDR of *AoamyA*, *AoamyB*, *AoamyC*) |
| TamyB-F | CATCATCATCATCATCATTAAAGGGTGGAGAGTATATGATG (Primer for amplifying the terminator sequence (including Histag sequence) of *AoamyB*) |
| TamyB-R | TAGCCGGAGATTTGACCGAATC (Primer for amplifying the terminator sequence (including Histag sequence) of *AoamyB*) |
| Ade-TamyB-F | TTCGGTCAAATCTCCGGCTACTCATGCCGTCATGTCCAG (Primer for amplifying wild type *AoadeA* as the selectable marker) |
| Ade-R | TCTGCGCAACAGCATACGAG (Primer for amplifying wild type *AoadeA* as the selectable marker) |
| RHA-Ade-F | TCGTATGCTGTTGCGCAGACGGCCAAGAACAGCACTAC (Primer for amplifying the 3′ flanking region for HDR of *AoamyA*, *AoamyB*, *AoamyC*) |
| RHA-T-R | CTGCAGGTCGACGATCTCTAGACACGAGCTACTACAGATCTTG (Primer for amplifying the 3′ flanking region for HDR of *AoamyA*, *AoamyB*, *AoamyC*) |
| AcOS-F | AAACCCCACAGAAGGCATTTATGGCCTGCAAGTACAGCACC (Primer for amplifying AcOS gene optimized for *A. oryzae* and verification of *AoamyA* deletion) |
| AcOS-R | TTAATGATGATGATGATGATGGACCTTCAGCAGCTCGAGCAT (Primer for amplifying AcOS gene optimized for *A. oryzae* and verification of *AoamyB* and *AoamyC* deletion) |
| ΔamyB-F | GGCAACAAGCATGGAGTC (Primer for verification of *AoamyB* deletion) |
| AdeA-F | CTCATGCCGTCATGTCCAG (Primer for verification of *AoamyB* and *AoamyC* deletion) |
| ΔamyB-R  (ΔamyC-R) | GCCATTTCGTCAAGGTATAG (Primer for verification of *AoamyB* and *AoamyC* deletion) |
| ΔamyC-F | GATGCAGAGACGGATGGTTG (Primer for verification of *AoamyC* deletion) |
| ΔamyBC-R | CTGCCATGTTCCACCACAG (Primer for verification of *AoamyB* and *AoamyC* deletion) |
| Δamy-F | GACGGCCTCCGTATCGAC (Primer for verification of *AoamyA* deletion) |

**Table S2 Predicted off-target sites**

| **Target** | **Chromosome** | **Position** | **Direction** | **Mismatches** | **Bulge Size** |
| --- | --- | --- | --- | --- | --- |
| GGCGAGGGCGGCAACATCGTNGG  GGCGAtGGCGGCgcCATCGTGGG | Scaffold 4 | 1394215 | - | 3 | 0 |
| GGCGAGGGCGGCAACATCGTNGG  GGCGAcGGCGGCggCATCGTCGG | Scaffold 7 | 992991 | + | 3 | 0 |
| GGCGAGGGCGGCAACATCGTNGG  GGCGAcGaCGaCAACATCGTGGG | Scaffold 20 | 161123 | - | 3 | 0 |
| GGCGAGGGCGGCAACATCGTNGG  GGCGAGGGCGGCtgCgTCGTTGG | Scaffold 20 | 252649 | - | 3 | 0 |
